# The Bioavailability of Xanthohumol in Humans and the Influence of Formulation and Dose: Randomized Controlled Trial Data

**DOI:** 10.1002/mnfr.70413

**Published:** 2026-02-22

**Authors:** Sara Brehmer‐Henkel, Christina Diekmann, Marit Eickeler, Ronja Maris, Christina Kopp, Martin Coenen, Robert Németh, Birgit Stoffel‐Wagner, Nadine Sus, Jan Frank, Sarah Egert

**Affiliations:** ^1^ Institute of Nutritional and Food Sciences Nutritional Physiology University of Bonn Bonn Germany; ^2^ Clinical Study Core Unit Study Center and Central Laboratory Bonn Institute of Clinical Chemistry and Clinical Pharmacology University Hospital Bonn Bonn Germany; ^3^ Institute of Medical Biometry Informatics and Epidemiology Venusberg‐Campus 1 University Hospital Bonn Bonn Germany; ^4^ Department of Food Biofunctionality (140b) Institute of Nutritional Sciences Hohenheim University Stuttgart Germany

**Keywords:** bioactivity, bioavailability, blood pressure, energy expenditure, flavonoid, xanthohumol

## Abstract

Xanthohumol is a prenylated chalcone, which is mainly found in hops. Experimental studies show a variety of cardioprotective effects for xanthohumol, but so far there are only a few controlled studies on the bioavailability and efficacy of xanthohumol in humans. In a randomized crossover bioavailability trial, the plasma kinetics of 86 mg and 172 mg each of micellar or native xanthohumol were investigated. Blood samples were obtained at fasting (*t*_0_), regularly until 9 h and 24 h after oral xanthohumol bolus administration and the plasma concentrations of xanthohumol, xanthohumol glucuronide, and xanthohumol sulfate were analyzed. Micellation increased the area under the plasma concentration–time curve (AUC) (*p* < 0.001) and maximum plasma concentration (*p* <0.001). Both formulations showed a dose‐dependent increase in xanthohumol maximum concentrations and AUC. The bioavailability was mainly influenced by micellation (*p* <0.001) and was approx. 9‐fold higher after intake of micellar xanthohumol than after intake of the native form, irrespective of the dose (*p* = 0.480). A subsequent randomized placebo‐controlled crossover bioactivity trial showed no acute effects of 172 mg micellar xanthohumol on resting energy expenditure, blood pressure, or heart rate. To conclude, bioavailability was significantly higher after ingestion of micellar xanthohumol than after ingestion of native xanthohumol.

## Graphical abstract

The effects of two different doses of micellar and native xanthohumol on plasma kinetics were investigated in 12 healthy men and women. Micellization significantly improved the bioavailability of xanthohumol. A subsequent placebo‐controlled study on the acute effects of xanthohumol on resting energy expenditure, blood pressure, and heart rate in 16 healthy women showed no significant differences in the parameters between the groups.

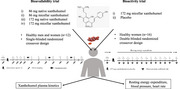

**Abbreviations**
AUC: Area under the curve
BP: Blood pressure
*C*_max_
: Maximum plasma concentration of xanthohumol
DBP: Diastolic blood pressure
HR: Heart rate
*k*
: Elimination rate constant
METS: Metabolic equivalent of tasks
REE: Resting energy expenditure
RQ: Respiratory quotient
SBP: Systolic blood pressure
*t*_1/2_
: Elimination half‐time
*t*_max_
: Timepoint of maximum plasma concentration of xanthohumol
VCO_2_
: Exhaled carbon dioxide volume
VO_2_
: Inhaled oxygen volume

## 1 Introduction

Xanthohumol is a prenylated chalcone that can be found at low concentrations in hops, which is why intake of xanthohumol via food is only possible in small quantities [1, 2, 3]. Intestinal absorption and consequently the bioavailability of many flavonoids are limited due to their hydrophobic properties [4, 5, 6]. Only a limited amount of data from animal models and human studies is available on the bioavailability of xanthohumol. These data indicate that xanthohumol is poorly absorbed and rapidly metabolized and excreted, which limits its bioavailability [7, 8]. Metabolization of xanthohumol is characterized by its rapid isomerization to isoxanthohumol, which presumably occurs mostly in the gastrointestinal tract. In the systemic circulation, 96% of xanthohumol and 100% of its metabolites are present as conjugates [9], of which over 90% are glucuronides [10]. Bioavailability is a prerequisite for any physiological effects of xanthohumol in humans.

A pilot study investigated the plasma kinetics of major human xanthohumol metabolites in five healthy volunteers following the administration of 43 mg micellar or native xanthohumol [11]. Xanthohumol‐7‐*O*‐glucoronide and xanthohumol‐4′‐*O*‐sulfate were quantitatively the most important metabolites. Smaller quantities of mixed metabolites containing sulfates and glucuronides as well as double glucuronide conjugates of xanthohumol and derived prenylated chalcones and flavonoids were also found. Due to the complex and time‐consuming analysis required to quantify xanthohumol metabolites, this study was limited to a small number of participants and the obtained plasma kinetics thus require confirmation in a larger trial [11].

In addition to the abovementioned trial, only a few studies have tested the effects of xanthohumol in humans in terms of glucose and lipid metabolism, which failed to show any significant results [12, 13, 14]. By contrast, reductions of cardiometabolic risk parameters in glucose and lipid metabolism have been demonstrated in animal models [15, 16]. Furthermore, xanthohumol was reported to enhance weight loss [15], which indicates an increase in energy metabolism. In vitro, xanthohumol increases energy turnover by “beiging” white adipocytes [17] and promotes mitochondrial uncoupling in myocytes [18]. In addition, xanthohumol acts as a ligand for bile acid receptors that regulate energy metabolism [19]. No experimental study has investigated the effects of xanthohumol on resting energy expenditure (REE) or other metabolic parameters such as blood pressure (BP) and heart rate (HR) in humans or animal models.

No study has applied different doses of xanthohumol in different formulations and defined the resulting plasma kinetics and apparent bioavailability in human models. In addition, it is unclear whether there are sex‐specific differences in the bioavailability and metabolism of xanthohumol. Therefore, this research project aimed to systematically investigate the bioavailability of xanthohumol using two formulations (native and micellar) and two doses (86 and 172 mg) (bioavailability trial). In a subsequent placebo‐controlled study (bioactivity trial), we examined the short‐term effects of xanthohumol on REE, BP, and HR. Both studies were conducted in metabolically healthy participants.

## 2 Participants and Methods

### 2.1 General Information on the Bioavailability and Bioactivity Trials

The studies were conducted according to the guidelines laid down in the 1964 Declaration of Helsinki and its later amendments, and all procedures involving human participants were approved by the ethics committee of the Medical Faculty of the Rheinische Friedrich–Wilhelms Universität Bonn, Germany (approval numbers: 242/22 and 486/22). Written informed consent was obtained from all participants prior to the commencement of the studies. The studies were conducted at the Institute of Nutritional and Food Sciences, Nutritional Physiology, University of Bonn. Both trials were registered at https://clinicaltrials.gov/ (NCT05524714 and NCT05711212).

### 2.2 Xanthohumol Capsules

The xanthohumol and placebo capsules used in these research projects were specifically produced for the purpose of these studies by AQUANOVA AG (Darmstadt, Germany). Micelle structures were produced with NovaSOL technology (AQUANOVA AG). Hard or soft gelatine capsules were filled with native or micellar solubilized xanthohumol. Each capsule contained 43 mg xanthohumol (native or micellar). Placebo capsules were soft gelatine capsules containing polysorbate 80 (E433). The entire production process was compliant with good manufacturing practice.

### 2.3 High‐Performance Liquid Chromatography Analyses of Xanthohumol

#### 2.3.1 Sample Preparation

Xanthohumol was analyzed in duplicate as previously described [20] with small modifications. Plasma samples were thawed on ice and 900 µL were transferred to a fresh glass tube, 10 µL ascorbic acid (150 mg in 1.5 mL H_2_O) and 3 mL acetonitrile/methanol (9:1, v:v) were added and vortex‐mixed for 3 min and centrifuged at 1690 × *g* for 5 min at 4°C. A volume of 2.7 mL of the supernatant was transferred to a fresh glass tube. A second extraction with 4 mL ethyl acetate and mixing for 20 min on a vertical rotator was followed by a centrifugation at 1690 × *g* for 5 min at 4°C and 3.6 mL of the supernatant was transferred. The pooled supernatants were evaporated to dryness at 10 mbar using a RVC 2‐33 IR centrifugal evaporator (Martin Christ Gefriertrockungsanlagen GmbH, Osterode am Harz, Germany). Samples were resuspended in 100 µL methanol/water (9:1, v:v) and 40 µL was injected into the HPLC. The extraction was optimized prior to analysis with spiked plasma and tested with plasma from a pilot trial (see High‐Performance Liquid Chromatography for information regarding the performance of the method).

#### 2.3.2 High‐Performance Liquid Chromatography

Xanthohumol and its metabolites were chromatographed on a JASCO HPLC system (LC Net II ADC, AS‐2059‐SF Plus, PU‐2080 Plus, Intelligent Column Thermostat CO‐2060 Plus, DG‐2080‐53, LG‐2080‐02, and a photodiode array detector PDA MD‐2018; JASCO, Groβ‐Umstadt, Germany) equipped with a Kinetex PFP column (Kinetex PFP, 100 Å pore size, 250 × 4.6 mm (length × diameter), 5 µm particle size; Phenomenex) maintained at 35°C. Mobile phase A (deionized water with 5% formic acid) and mobile phase B (acetonitrile with 10% deionized water and 5% formic acid) were delivered at a flow rate of 0.8 mL/min following a multistep gradient method (0 min 0% B, 5 min 20% B, 4 min 30% B, 10 min 30% B, 15 min 75% B, 22 min 100% B, and 26 min 30% B). The eluent was monitored using a photodiode array detector set to 292 nm (for isoxanthohumol glucuronide, isoxanthohumol sulfate, isoxanthohumol, 8‐prenylnaringenin, and 6‐prenylnaringenin) and 365 nm (for xanthohumol glucuronide, xanthohumol sulfate, and xanthohumol) with spectra obtained over the 200–850 nm range. Peaks were recorded and integrated using ChromNav version 2 (JASCO), identified by retention time and spectra and quantified by comparing the peak areas to those of authentic external standards. Standard curves were constructed from 0.5 to 25 µg/mL, and linearity was given for all compounds *R* ^2^ < 0.985. Standards for xanthohumol, isoxanthohumol, 6‐prenylnaringenin, and 8‐prenylnaringenin were purchased from PhytoLab GmbH & Co. KG (Vestenbergsgreuth, Germany). Standards for metabolites were synthesized at the Technical University Munich according to a developed protocol [21].

Recovery, accuracy, and precision of the adapted method were determined using plasma spiked with low and high amounts of all analytes (2.5 and 20 µg/mL, respectively) and extracted in triplicate on two independent days. Recovery was ≥85% for all compounds at both low and high concentrations. Inter‐ and intraday accuracy was within ≤16% of the known concentrations at the low and ≥12% at the high concentrations tested. Inter‐ and intraday precision was high with ≤10% variation at the low and ≤6% variation at the high concentrations of all analytes.

#### 2.3.3 Determination of the Intake of Energy and Nutrients, Physical Activity, and Nutritional Status

Dietary records were analyzed using the computer‐based nutrient calculation program EBISpro (University of Hohenheim, Stuttgart, Germany), based on the German Nutrient Database Bundeslebensmittelschlüssel version II.3 (Max‐Rubner Institute, Karlsruhe, Germany). For activity records, the metabolic equivalent of tasks (MET) was calculated [22].

Body height was determined on a stadiometer to the nearest 0.1 cm in both trials. In the bioavailability trial, body weight was determined to the nearest 100 g. Body composition was measured using bioelectrical impedance analysis (Nutrigard‐M, Multi Frequency Phase‐Sensitive Bioelectrical Impedance Analyzer, Data Input). Fat‐free mass was calculated as described by Sun et al. [23]. Fat mass was calculated by subtracting fat‐free mass from total body weight. In the bioactivity trial, body weight and body composition (fat mass and fat‐free mass) were determined via air‐plethysmography (BODPOD system, COSMED Srl.).

## 3 Bioavailability Trial

### 3.1 Participants

**FIGURE 1:**
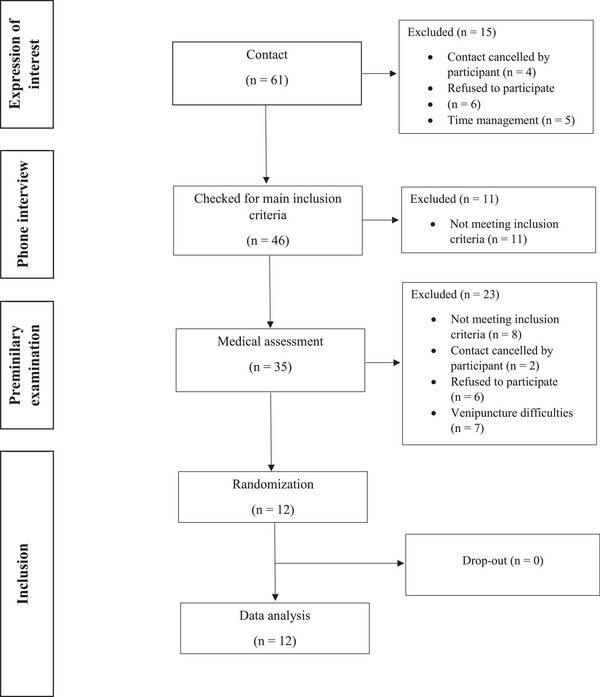
Flowchart of participants (bioavailability trial).

Participants were recruited from the University of Bonn by flyers and announcements in university courses. In total, 46 interested persons participated in a telephone anamnesis (including medical history and dietary habits) and 35 attended a clinical and physical assessment. Seventeen participants met all the inclusion criteria and provided written consent to participate in the study. Finally, 12 non‐smoking, normal‐weight, Caucasian men and women aged 20–30 years were randomized (Figure 1). The participants were in good health, as determined by a basic examination (body weight and height, BP, HR, medical anamnesis, and fasting blood analyses; baseline characteristics are presented in Table 1). Exclusion criteria were overweight or obesity, metabolic and endocrine diseases, malabsorption syndromes, smoking, pregnancy or lactation, alcohol abuse, and use of dietary supplements or any form of medication. All participants were asked to maintain their regular lifestyle and extent of physical activities throughout the study.

**TABLE 1:** Baseline characteristics of participants (bioavailability trial).

	Total (*n* = 12)	Men (*n* = 6)	Women (*n* = 6)
Age (years)	23.9 ± 3.3	24.2 ± 3.7	23.7 ± 3.3
Body height (cm)	1.71 ± 0.06	1.72 ± 0.06	1.70 ± 0.06
Body weight (kg)	63.4 ± 5.5	65.4 ± 6.4	61.5 ± 4.1
BMI (kg/m^2^)	21.7 ± 1.5	22.0 ± 1.6	21.4 ± 1.6
Fat‐free mass (kg)	49.2 ± 8.1	56.7 ± 8.0*	44.2 ± 3.0
Fat mass (%)	20.7 ± 9.4	13.6 ± 4.6*	27.8 ± 7.3
Waist circumference (cm)	74.4 ± 5.0	77.3 ± 5.0**	71.5 ± 3.2
Systolic BP (mmHg)	121± 8	125 ± 8**	116 ± 4
Diastolic BP (mmHg)	77 ± 7	79 ± 7	74 ± 6
Heart rate (min^−1^)	65 ± 11	66 ± 12	64 ± 11
Serum triglycerides (mg/dL)	75 ± 23	76 ± 26	74 ± 23
Serum cholesterol (mg/dL)	153 ± 31	148 ± 17	158 ± 43
Serum LDL cholesterol (mg/dL)	83 ± 28	79 ± 22	87 ± 34
Serum HDL cholesterol (mg/dL)	60 ± 10	56 ± 6	63 ± 13
Plasma glucose (mg/dL)	82 ± 6	84 ± 7	80 ± 4
Serum uric acid (mg/dL)	4.75 ± 1.11	5.65 ± 0.66***	3.85 ± 0.59
Serum creatinine (mg/dL)	0.83 ± 0.15	0.96 ± 0.09***	0.71 ± 0.08
Serum gamma GT (U/L)	12.9 ± 3.1	13.8 ± 3.1	12.0 ± 3.0
Serum ALT (U/L)	21.5 ± 16.4	26.2 ± 22.9	16.8 ± 3.5
Serum AST (U/L)	21.0 ± 7.9	21.8 ± 10.6	20.2 ± 4.9
Hematocrit (%)	42 ± 4	45 ± 3*	39 ± 4
Blood Hb (g/L)	14.1 ± 1.7	15.4 ± 0.8*	12.7 ± 1.3

*Note*: Data represent mean ± SD.

Abbreviations: ALT, alanine aminotransferase; AST, aspartate aminotransferase; BP, blood pressure; GT, glutamyl transferase; Hb, hemoglobin.

* *p* < 0.01

** *p* < 0.05

*** *p* < 0.001 compared with women (according to the unpaired *t*‐test)

### 3.2 Study Design

The study was conducted as a single‐blind (participants), randomized four period, four treatment crossover study (Williams‐design) between November 2022 and January 2023. Participants refrained from ingesting (poly)phenol‐containing dietary supplements, beer, and other hops‐containing beverages for 4 weeks before and during the entire study period and documented their habitual diet on 4 consecutive days before the study. Additionally, the participants completed 1‐day dietary and activity records on the 4 days prior to the respective intervention days.

On intervention days, participants visited the study site in the morning after a 12‐h overnight fast and control parameters, such as BP and HR (Boso Carat Professional; Bosch + Sohn GmbH, Jungingen, Germany), body weight, and body composition were measured. Each subject underwent one of four interventions in a random order, separated by 14‐day washout periods. The treatments comprised of capsules containing i) 86 mg micellar xanthohumol, ii) 172 mg micellar xanthohumol, iii) 86 mg native xanthohumol, and iv) 172 mg native xanthohumol, which were ingested together with 200 mL of water.

Two hours after administration of xanthohumol, a standardized breakfast (with 300 mL water, white bread, butter, cheese, and curd cheese) and 5 h after ingestion, a standardized lunch (pasta with cheese sauce and parmesan), and 8 h after ingestion, a snack (packaged lemon cake) was served. A standardized dinner (white bread, butter, cheese, and egg) was taken home. All foods were (poly)phenol‐free, weighed to the nearest gram, and satisfied the energy demand of each participant. Participants were allowed to drink only water during each study day.

Blood samples were collected before (baseline) and 30, 60, 90, 120, 180, 300, 360, 420, 480, 540, and 1440 min after xanthohumol administration. Blood was obtained using an indwelling cannula for samples collected up to 9 h and thereafter by venipuncture. Blood was drawn into tubes containing potassium EDTA (Sars‐tedt, Nuembrecht, Germany) and immediately centrifuged (3000 × *g*, 15 min, 8°C). Plasma was stored at −80°C until analyses.

Additionally, fasting blood was collected to determine hematological markers (including counts of erythrocytes, leukocytes, and thrombocytes as well as hemoglobin, hematocrit, mean corpuscular volume, mean corpuscular hemoglobin, and mean corpuscular hemoglobin concentration) in fresh blood samples. Blood count parameters were analyzed by fluorescence flow cytometry, the resistance measurement technique, and photometry using a hematology analyzer (Sysmex XN9000 and Sysmex XN10; Sysmex, Norderstedt, Germany). All analyses were conducted without knowledge of the treatment groups.

#### 3.2.1 Plasma Kinetic Calculations

Xanthohumol, xanthohumol glucuronide, and xanthohumol sulfate were analyzed and the sum of all was defined as total xanthohumol. Additionally, isoxanthohumol, isoxanthohumol sulfate and their sum (total isoxanthohumol), and 8‐prenylnaringenin were analyzed. Maximum plasma xanthohumol concentrations (*C*_max_) and times to achieve maximum plasma concentrations (*t*_max_) were obtained by visually inspecting each participant's plasma concentration profile. The area under the plasma concentration–time curve (AUC) was determined using the linear trapezoidal rule.

The slope of the terminal log‐linear portion of the concentration–time profile was determined using least‐squares regression analyses and used as the elimination rate constant (*k*). The terminal elimination half‐life (*t*_1/2_) was calculated as ln 2/*k*. The rate of elimination, *k*, and *t*_1/2_ values of plasma xanthohumol could not be determined for individual concentration–time curves because too few timepoints were available during the elimination phase. Therefore, these kinetic parameters were calculated only from the mean value curves obtained in response to the four xanthohumol treatments.

In addition, the estimated absorption of xanthohumol (apparent bioavailability) was calculated by first multiplying *C*_max_ determined in the plasma kinetic study by an estimate of the participant's plasma volume according to Nadler et al. and then dividing this value by the dose administered [24].

#### 3.2.2 Statistical Analyses

All statistical analyses were performed using SPSS version 29 (SPSS Inc., Chicago, USA). All differences were considered significant at *p* < 0.05 (two‐tailed). Results are reported as mean values and SEM unless otherwise indicated.

Univariate linear mixed models were used to investigate the effect of the dose and formulation of xanthohumol for *C*_max_, *t*_max_, and AUC (for xanthohumol glucuronide, xanthohumol sulfate, xanthohumol, total xanthohumol, isoxanthohumol, isoxanthohumol sulfate, total isoxanthohumol, and 8‐prenylnaringenin) in bioavailability trial and REE, respiratory quotient (RQ), resting BP, and HR in bioactivity trial.

In the bioavailability trial, the respective parameters were set as the dependent variable. Subject ID, visit, formulation, dose, and sex were set as fixed factors, formulation and dose were set as interacting fixed factors, and subject ID was set as a random factor. Model residuals were plotted as QQ to test if they were normally distributed. Since this was not the case, the model was then recalculated with logarithmized (log_10_) data. To analyze differences in xanthohumol plasma concentration (for xanthohumol glucuronide, xanthohumol sulfate, xanthohumol, and total xanthohumol) between timepoints, linear mixed models were used. Plasma concentration (xanthohumol glucuronide, xanthohumol sulfate, xanthohumol, and total xanthohumol) was set as the dependent variable. Subject ID and visit were set as subjects and timepoints were set as a repetition factor. Visit, timepoints, and treatment were set as fixed factors, timepoints and treatment were set as interacting fixed factors, and subject ID and visit were set as random factors. Model residuals were plotted as QQ to test if they were normally distributed. The model was then recalculated with logarithmized (log_10_) data. To calculate differences in *C*_max_, *t*_max_, and AUC for total xanthohumol, xanthohumol glucuronide, xanthohumol sulfate, and xanthohumol, linear mixed models were used. The respective parameters were set as the dependent variable. Subject ID was set as the subject, visit, metabolite, treatment, sex, and subject ID were set as fixed factors, and treatment and total xanthohumol, xanthohumol glucuronide, xanthohumol sulfate, and xanthohumol were set as interacting fixed factors. Subject ID was set as a random factor. Model residuals were plotted as QQ to test if they were normally distributed. The model was then recalculated with logarithmized (log_10_) data. The sample size of *n* = 12 for the bioavailability trial was determined upon recommendation with the regulations for bioequivalence studies and based on previous bioavailability trials of our groups [25, 26, 27]. Significant differences of baseline characteristics between sexes were analyzed using the paired *t*‐test. Differences in diet and activity between the days before the intervention and differences in body weight, body composition, and resting BP and HR between each intervention morning were analyzed using a linear mixed model, with visit and sex set as fixed factors and interacting fixed factors. Subject ID was set as a random factor.

### 3.3 Bioactivity Trial

#### 3.3.1 Participants

**FIGURE 2:**
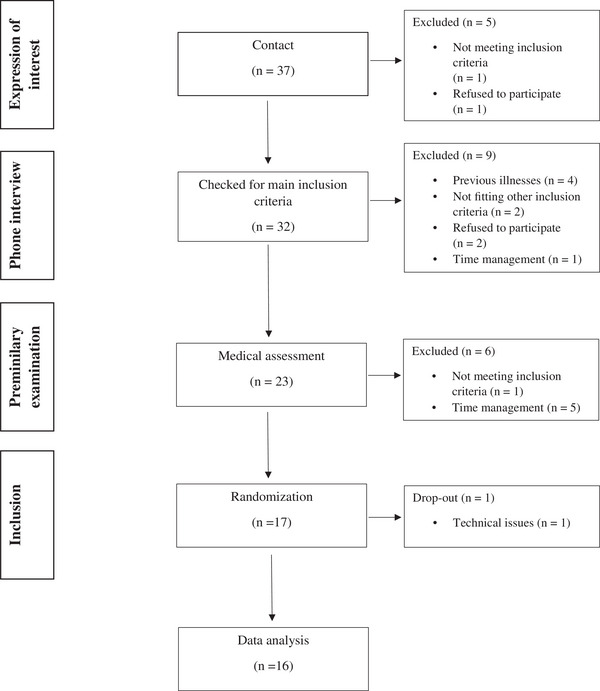
Flowchart of participants (bioactivity trial).

Participants were recruited from the University of Bonn via announcements in university courses. In total, 32 interested women participated in a telephone anamnesis (including medical history and dietary habits), and 23 attended a clinical and physical assessment. Seventeen participants met all the inclusion criteria and provided written consent to participate in the study, one of whom dropped out due to technical issues (Figure 2). Finally, data of 16 non‐smoking, normal‐weight, Caucasian women aged 20–30 years were analyzed. The participants were in good health, as determined by a basic examination (body weight and height, BP, HR, medical anamnesis, and fasting blood analyses; baseline characteristics are presented in Table 2). Exclusion criteria were overweight or obesity, metabolic and endocrine diseases, malabsorption syndromes, smoking, pregnancy or lactation, and alcohol abuse or use of any form of medication (except oral contraceptives). All participants were asked to maintain their regular lifestyle and usual extent of physical activities throughout the study.

**TABLE 2:** Baseline characteristics of participants (bioactivity trial).

	Women (*n* = 16)
Age (years)	23.4 ± 2.8
Body height (cm)	1.68 ± 0.08
Body weight (kg)	59.2 ± 6.8
BMI (kg/m^2^)	20.7 ± 1.3
Fat‐free mass (kg)	44.5 ± 4.5
Fat mass (%)	24.6 ± 4.1
Waist circumference (cm)	73.1 ± 6.0
Systolic BP (mmHg)	115 ± 8
Diastolic BP (mmHg)	74 ± 6
Heart rate (min^−1^)	69 ± 9
Resting energy expenditure (kcal/24 h)	1434 ± 146
Serum triglycerides (mg/dL)	71 ± 21
Serum cholesterol (mg/dL)	159 ± 29
Serum LDL cholesterol (mg/dL)	86 ± 19
Serum HDL cholesterol (mg/dL)	64 ± 14
Plasma glucose (mg/dL)	81 ± 5
Thyroid‐stimulating hormone (µU/mL)	1.62 ± 0.84
Serum creatinine (mg/dL)	0.72 ± 0.08
Serum gamma GT (U/L)	11.6 ± 4.8
Serum ALT (U/L)	15.9 ± 5.9
Serum AST (U/L)	17.6 ± 3.8
Hematocrit (%)	39 ± 2
Blood Hb (g/L)	13.0 ± 0.8

*Note*: Data represent mean ± SD.

Abbreviations: ALT, alanine aminotransferase; AST, aspartate aminotransferase; BP, blood pressure; GT, glutamyl transferase; Hb, hemoglobin.

#### 3.3.2 Study Design

The study was conducted in a double‐blinded, randomized, placebo‐controlled crossover design. For each treatment (xanthohumol and placebo), one main examination day and one post‐examination day (24 h after ingestion) were set. All women were tested in the same phase of their menstrual cycle or took ovulation‐suppressing oral contraceptives (*n* = 6) to avoid an effect of the menstrual cycle on REE. For this reason, the interventions took place at an individually defined interval of 4 weeks.

On examination days, participants arrived at the study unit in the morning after a 12‐h overnight fast. Body weight and body composition were determined. Then, a 30‐min baseline measurement of REE was performed and resting BP and HR were measured once. After a 5‐min break during which xanthohumol capsules were ingested, REE was determined for 3 h. Resting BP and HR were measured every 15 min. On the following day, 24 h after capsule intake, a follow‐up respiratory measurement was performed for 30 min. In addition, BP and HR were measured. All measurements were performed by the same trained investigator.

The same xanthohumol capsules were used as in bioavailability trial. They each contained 43 mg solubilized xanthohumol and were administered in the micellar formulation. In terms of the pharmacokinetics of xanthohumol, 172 mg micellar xanthohumol has the highest apparent bioavailability and was safe and well tolerated; therefore, this dose and formulation were selected in the bioactivity trial. Participants ingested four capsules (43 mg xanthohumol each or placebo) with 200 mL tap water. Xanthohumol is mostly absorbed within 1 h of ingestion (see results of bioavailability trial). Therefore, an investigation period of 3 h was selected.

Participants abstained from medications other than habitual medication (contraceptives) and from alcohol and energetic physical activity for 24 h before the respiratory measurements. To limit diurnal variation and inter‐ and intra‐subject variations, all measurements were performed according to an identical schedule and at the same time of day. Participants were instructed to come to the metabolic ward of the institute by car or bus.

Participants refrained from ingesting (poly)phenol‐containing dietary supplements, beer, and other hop‐containing beverages 2 days before the examination days. Additionally, participants documented their habitual diet on 4 consecutive days (3 days before examination days and on the examination day). Furthermore, they completed 2‐day activity records the day before and on the examination day.

#### 3.3.3 Measurements

##### 3.3.3.1 Resting Energy Expenditure (REE), Blood Pressure (BP), and Heart Rate (HR)

REE and substrate oxidation were determined by gas analysis. Inhaled oxygen volume VO_2_ and exhaled carbon dioxide volume VCO_2_ were determined every 10 s using a ventilated canopy system (Quark‐RMR; COSMED Srl., Rome, Italy). A mass flow sensor measured volume and airflow. Flow and gas analyzers were calibrated before each intervention measurement. VO_2_ and VCO_2_ were converted to REE using the abbreviated Weir equation [28]. Substrate oxidation was determined by the RQ of VCO_2_ and VO_2_.

BP and HR were measured under standardized conditions in accordance with European and American guidelines [29, 30]. BP was measured on the upper arm with a clinically validated BP monitor according to the method of Riva Rocci and Korotkow [31] (Boso Carat Professional, Bosch + Sohn GmbH). The measurements were performed in duplicate. If the two measurements differed by more than 10 mmHg (BP) or 10 bpm (HR), a third measurement was taken. Finally, the mean value was used for statistical analysis.

#### 3.3.4 Statistical Analyses

In the bioactivity trial, participant ID and visit were set as subjects, and timepoints were set as repeated measures. The respective parameters were set as the dependent variable, and visit, timepoints, and treatment were set as factors. The baseline (*t*_0_) of the respective parameters was set as a covariate. Timepoints and treatment were set as interacting fixed factors, and subject ID and visit were set as random factors. During data processing of indirect calorimetry, data from the first 5 min were removed. VO_2_ and VCO_2_ were determined every 10 s for 30 min (baseline) or 185 min (study). An outlier was defined as when the carbon dioxide content under the canopy was below 0.7% or above 1.1%, which corresponds to the acceptance range specified by the device manufacturer. Outliers were removed using the dual control principle. Subsequently, mean VO_2_ and VCO_2_ values were used to calculate mean REE and RQ for statistical analyses. Mean REE and RQ were calculated in 15‐min intervals to compare timepoints. Differences in diet and activity between the examination day and post‐examination and differences in habitual diet, body weight, and body composition between treatments were analyzed using the paired *t*‐test. All differences were considered significant at *p* < 0.05 (two‐tailed). Results are reported as mean values and SEM unless otherwise indicated.

## 4 Results

### 4.1 Control Variables of Both Bioavailability and Bioactivity Trials

No adverse effects of taking xanthohumol were reported. Habitual daily energy and nutrient intakes did not significantly differ between the interventions. Physical activity and energy and nutrient intake did not significantly differ between the evening before the intervention and on intervention days and between different interventions. Additionally, body weight, body composition, BP, and HR did not significantly differ between the intervention days either (Table S1 for bioavailability trial, data not shown for bioactivity trial). This was true for both sexes in the bioavailability trial.

### 4.2 Bioavailability Trial

#### 4.2.1 Plasma Kinetics of Xanthohumol

**FIGURE 3:**
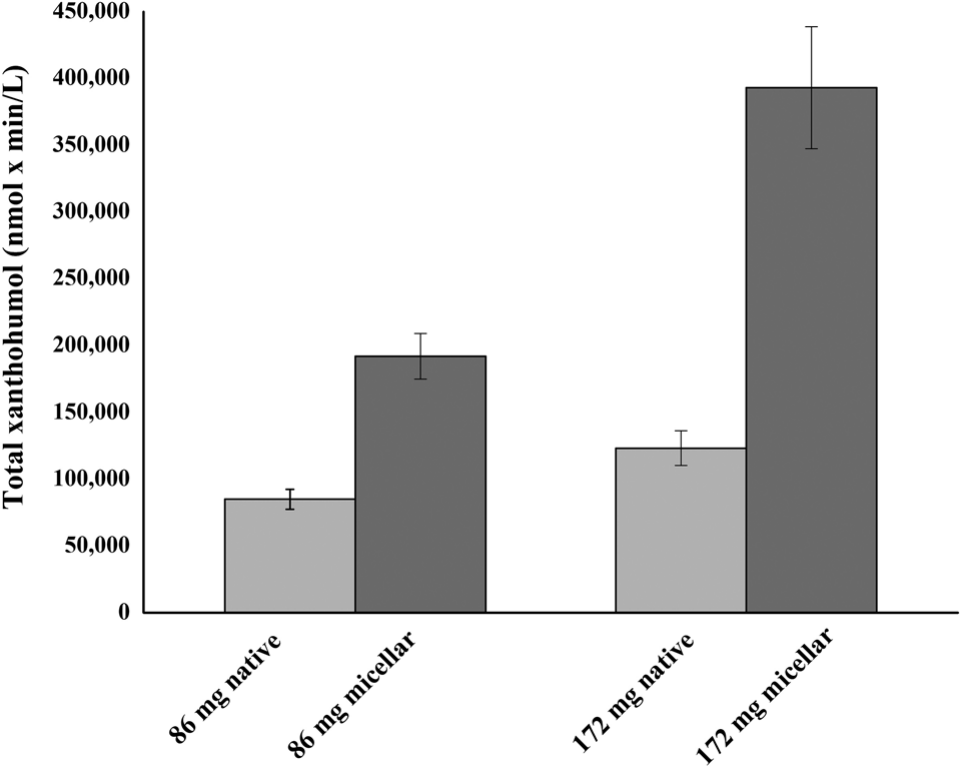
AUC in metabolically healthy men and women after a single oral dose of micellar xanthohumol (86 and 172 mg) or native xanthohumol (86 and 172 mg) (*n* = 12). Values represent mean ± SEM.

**FIGURE 4:**
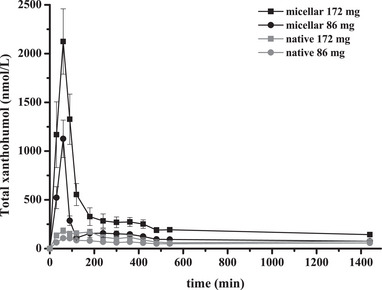
Plasma xanthohumol concentration–time curves in metabolically healthy men and women after a single oral dose of micellar xanthohumol (86 and 172 mg) or native xanthohumol (86 and 172 mg) (*n* = 12). Values represent mean ± SEM.

The relative AUC of micellar xanthohumol compared to native xanthohumol was 235 ± 19% for 86 mg dose xanthohumol and 320 ± 44% for 172 mg dose xanthohumol (95% CI: 0.374; 0.488, *p* < 0.001) (Figure 3). Micellation significantly increased the AUC for total xanthohumol (95% CI: 0.374; 0.488), xanthohumol glucuronide (95% CI: 0.379; 0.494), xanthohumol sulfate (95% CI: 0.103; 0.412) and xanthohumol (95% CI: 0.326; 0.721) (all *p* < 0.01). Additionally, micellation increased *C*_max_ of total xanthohumol (95% CI: 0.879; 1.055), xanthohumol glucuronide (95% CI: 0.885; 1.064), xanthohumol sulfate (95% CI: 0.304; 0.593), and xanthohumol (95% CI: 0.675; 0.972) (all *p* < 0.001) and micellation increased apparent bioavailability for total xanthohumol (95% CI: 0.874; 1.051, *p* < 0.001) (Table 3 and Figure 4). *T*_max_ was reached significantly faster for total xanthohumol (95% CI: −0.411; −0.163), xanthohumol glucuronide (95% CI: −0.423; −0.166), xanthohumol sulfate (95% CI: −0.505; −0.229), and xanthohumol (95% CI: −0.438; −0.207) (all *p* < 0.001) with the micellar formulation than with the native formulation. A higher dose increased AUC (total xanthohumol: 95% CI: 0.174; 0.288, xanthohumol glucuronide: 95% CI: 0.173; 0.288, xanthohumol sulfate: 95% CI: −0.068; 0.241, xanthohumol: 95% CI: 0.095; 0.490, *p* = 0.263 for xanthohumol sulfate, all other *p* < 0.01). Additionally, a higher dose increased *C*_max_ (total xanthohumol: 95% CI: 0.185; 0.361, xanthohumol glucuronide: 95% CI: 0.179; 0.358, xanthohumol sulfate: 95% CI: 0.103; 0.392, xanthohumol: 95% CI: 0.235; 0.532, all *p* < 0.01) (Table 3).

**TABLE 3:** Kinetic variables of plasma xanthohumol, xanthohumol glucuronide, xanthohumol sulfate, and total xanthohumol concentrations in metabolically healthy men and women after a single oral dose of micellar xanthohumol (86 or 172 mg) or native xanthohumol (86 or 172 mg).^a^

	Micellar xanthohumol (86 mg)	Micellar xanthohumol (172 mg)	Native xanthohumol (86 mg)	Native xanthohumol (172 mg)	*p*‐Dose effect	*p*‐Formulation effect	*p*‐Dose × formulation effect
**Plasma xanthohumol**							
AUC_0‐1440 min_	3869 ± 416	9978 ± 1661	1899 ± 460	3041 ± 692	0.005	<0.001	0.406
*C*_max_ (nmol/L)	20 ± 3	69 ± 9	5 ± 1	7 ± 1	<0.001	<0.001	0.024
*t*_max_ (min)	53 ± 5	60 ± 6	158 ± 28	105 ± 20	0.241	<0.001	0.039
**Plasma xanthohumol glucuronide**							
AUC_0‐1440 min_	184 080 ± 16 890	376 430 ± 47 142	80 134 ± 7047	116 552 ± 12 376	<0.001	<0.001	0.009
*C*_max_ (nmol/L)	1196 ± 160	2284 ± 285	128 ± 14	236 ± 26	<0.001	<0.001	0.799
*t*_max_ (min)	58 ± 5	63 ± 6	148 ± 34	135 ± 26	0.811	<0.001	0.823
**Plasma xanthohumol sulfate**							
AUC_0‐1440 min_	3953 ± 558	5562 ± 603	3022 ± 763	3601 ± 778	0.263	0.002	0.173
*C*_max_ (nmol/L)	16 ± 2	28 ± 3	7 ± 1	13 ± 4	0.001	<0.001	0.945
*t*_max_ (min)	53 ± 4	80 ± 17	164 ± 36	175 ± 34	0.278	<0.001	0.516
**Plasma total xanthohumol** ^a^							
AUC_0‐1440 min_	191 903 ± 16 977	392 984 ± 47 713	85 054 ± 7845	123 295 ± 12 978	<0.001	<0.001	0.007
*C*_max_ (nmol/L)	1228 ± 160	2379 ± 288	134 ± 14	249 ± 27	<0.001	<0.001	0.760
*t*_max_ (min)	58 ± 5	63 ± 6	148 ± 34	140 ± 33	0.898	<0.001	0.725
*t*_1/2_ (min)	602	502	1505	1003	—	—	—
Apparent bioavailability (*%*)^b^	1.28 ± 0.18	1.22 ± 0.14	0.14 ± 0.01	0.13 ± 0.01	0.480	<0.001	0.779

*Note*: Descriptive data are mean ± SEM. Statistic data is log_10_‐transformed.

Abbreviations: AUC, area under the plasma concentration‐time curve; *C*_max_, maximum plasma concentration; *t*_max_, time to reach the maximum plasma concentration; *t*_1/2,_ elimination half‐life.

^a^ The plasma total xanthohumol concentration was calculated as follows: plasma xanthohumol concentration (nmol/L) plus plasma xanthohumol glucuronide concentration plus plasma xanthohumol sulfate concentration (nmol/L).

^b^ Apparent bioavailability (*%)*, defined as the minimum absorbed amount of xanthohumol, was estimated by first multiplying the maximum total plasma concentration of xanthohumol by an estimate of the participant's mean plasma volume and then dividing this value by the dose administered.

The interindividual range of *C*_max_ of total xanthohumol was 565–2580 and 1084–4398 nmol/L for 86 and 172 mg micellar xanthohumol and 68–209 and 75–403 nmol/L for 86 and 172 mg native xanthohumol, respectively. Micellization increased *C*_max_ (mean) of total xanthohumol by at least 9‐fold for both doses. The interindividual range of *t*_max_ of total xanthohumol was 30–90 and 30–420 min for micellar and native xanthohumol, respectively. The interindividual variation coefficient of *C*_max_ of total xanthohumol was similar for the different treatments and was 34.5% for 86 mg native xanthohumol, 36.1% for 172 mg native xanthohumol, 40.1% for 172 mg micellar xanthohumol, and 43.1% for 86 mg micellar xanthohumol. The elimination half‐life of total xanthohumol was more than twice as long after administration of native (∼17–25 h) than after micellar xanthohumol (∼9–10 h). After 24 h, total xanthohumol concentrations of 57 nmol/L (86 mg native xanthohumol), 75 nmol/L (172 mg native xanthohumol), 76 nmol/L (86 mg micellar xanthohumol), and 144 nmol/L (172 mg micellar xanthohumol) were quantified in plasma. Apparent bioavailability of total xanthohumol was primarily influenced by micellization (*p* < 0.001) but was dose‐independent (*p* = 0.480). In addition, apparent bioavailability was more than 9‐fold higher (mean) for micellar xanthohumol than for native xanthohumol (micellar xanthohumol, range 0.54%–2.89%; native xanthohumol, range 0.04%–0.23%) (Table 3).

For all treatments, there was a significant time effect on the plasma concentrations of total xanthohumol, xanthohumol glucuronide, xanthohumol sulfate, and xanthohumol (all *p* < 0.001). *T*_max_ did not significantly differ between total xanthohumol, xanthohumol glucuronide, xanthohumol sulfate, and xanthohumol, and AUC (Table 3). Xanthohumol glucuronide was the predominant conjugate (over 94%) in all treatment groups. AUC and *C*_max_ were significantly higher for xanthohumol glucuronide than for xanthohumol sulfate and xanthohumol (all *p* < 0.001) (Figures S1–S3).

The maximum measured plasma concentrations of 8‐prenylnaringenin are below 0.5 µmol/L, the mean *C*_max_ was below 0.1 µmol/L for all interventions (Tables S2–S6). Mean total isoxanthohumol plasma concentrations were also below 0.5 µmol/L for all interventions. There was a significant effect for dose (*p* < 0.001) and formulation (*p* = 0.006), but there was no interaction effect (*p* = 0.348) (Table 4).

**TABLE 4:** Kinetic variables of plasma isoxanthohumol, isoxanthohumol sulfate, 8‐prenlynaringenin, and total isoxanthohumol concentrations in metabolically healthy men and women after a single oral dose of micellar xanthohumol (86 or 172 mg) or native xanthohumol (86 or 172 mg).^a^

	Micellar xanthohumol (86 mg)	Micellar xanthohumol (172 mg)	Native xanthohumol (86 mg)	Native xanthohumol (172 mg)	*p*‐Dose effect	*p*‐Formulation effect	*p*‐Dose × formulation effect
**Plasma isoxanthohumol**							
AUC_0‐1440 min_	6665 ± 6041	4678 ± 882	128 ± 123	1987 ± 1357	0.981	0.176	0.566
*C*_max_ (nmol/L)	16 ± 8	79 ± 16	2 ± 2	12 ± 5	<0.001	<0.001	0.013
**Plasma isoxanthohumol sulfate**							
AUC_0‐1440 min_	50 301 ± 13 363	73 148 ± 13 822	19 543 ± 68 686	61 114 ± 10 236	0.005	0.028	0.274
*C*_max_ (nmol/L)	128 ± 22	299 ± 38	83 ± 20	220 ± 31	<0.001	0.013	0.473
**Plasma total isoxanthohumol** ^a^							
AUC_0‐1440 min_	56 966 ± 18 187	86 616 ± 13 336	19 671 ± 5508	63 102 ± 11 056	0.006	0.019	0.579
*C*_max_ (nmol/L)	136 ± 25	329 ± 38	83 ± 20	226 ± 33	<0.001	0.006	0.348
**Plasma 8‐Prenylnaringenin**							
AUC_0‐1440 min_	13 325 ± 6927	16 669 ± 10 160	16 088 ± 11 155	13 631 ± 6452	0.850	0.754	0.987
*C*_max_ (nmol/L)	96 ± 39	66 ± 26	52 ± 32	73 ± 30	0.885	0.334	0.425

*Note*: Descriptive data are mean ± SEM.

Abbreviations: AUC, area under the plasma concentration‐time curve; *C*_max_, maximum plasma concentration.

^a^ The plasma total isoxanthohumol concentration was calculated as follows: plasma isoxanthohumol concentration (nmol/L) plus plasma isoxanthohumol sulfate concentration (nmol/L).

#### 4.2.2 Sex Differences

AUC, *C*_max_, *t*_max_, and the apparent bioavailability did not significantly differ between men and women for total xanthohumol (Table 5). Additionally, xanthohumol glucuronide, xanthohumol sulfate, and xanthohumol did not significantly differ between the sexes for any treatment (data not shown).

**TABLE 5:** Sex‐specific kinetic variables of total plasma xanthohumol concentrations in metabolically healthy men and women after a single oral dose of micellar xanthohumol (86 or 172 mg) or native xanthohumol (86 or 172 mg).^a^

	Micellar xanthohumol (86 mg)	Micellar xanthohumol (172 mg)	Native xanthohumol (86 mg)	Native xanthohumol (172 mg)
AUC_0‐1440 min_	m: 196 175 ± 31 142 f: 187 631 ± 17 060	m: 374 993 ± 70 554 f: 414 574 ± 63 510	m: 79 419 ± 12 238 f: 90 690 ± 9167	m: 102 344 ± 17 946 f: 144 246 ± 15 604
*C*_max_ (nmol/L)	m: 967 ± 143 f: 1490 ± 254	m: 2235 ± 364 f: 2524 ± 472	m: 127 ± 18 f: 141 ± 23	m: 206 ± 31 f: 291 ± 39
*t*_max_ (min)	m: 60 ± 8 f: 55 ± 5	m: 60 ± 8 f: 65 ± 9	m: 165 ± 38 f: 130 ± 59	m: 190 ± 57 f: 90 ± 21
Apparent bioavailability (%)^a^	m: 1.02 ± 0.15 f: 1.54 ± 0.30	m: 1.16 ± 0.19 f: 1.27 ± 0.24	m: 0.14 ± 0.02 f: 0.15 ± 0.03	m: 0.11 ± 0.02 f: 0.15 ± 0.02

*Note*: Descriptive data are mean ± SEM.

Abbreviations: AUC, area under the plasma concentration‐time curve; *C*_max_, maximum plasma concentration; f, female; m, male; *t*_max_, time to reach the maximum plasma concentration.

^a^ Apparent bioavailability (*%)*, defined as the minimum absorbed amount of xanthohumol, was estimated by first multiplying the maximum total plasma concentration of xanthohumol by an estimate of the participant's mean plasma volume and then dividing this value by the dose administered.

### 4.3 Bioactivity Trial

#### 4.3.1 Resting Energy Expenditure (REE) and Respiratory Quotient (RQ)

**FIGURE 5:**
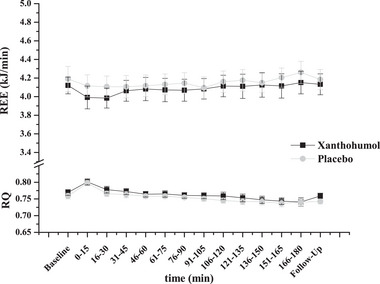
REE and RQ time curves in metabolically healthy women after a single oral dose of 172 mg micellar xanthohumol or placebo (*n* = 16). Values represent mean ± SEM.

REE did not significantly differ between the baseline, end of measurement, and follow‐up in either treatment group (time effect: *p* = 0.476; treatment effect: *p* = 0.911; time × treatment interaction: *p* = 0.853). RQ at the beginning of the 3‐h measurement was 0.80 ± 0.01 for both xanthohumol and placebo and decreased slowly and to a similar extent for both treatments (end of measurement: 0.74 ± 0.01 for both treatments; time effect: *p* < 0.001; treatment effect: *p* = 0.742; time × treatment interaction: *p* = 0.848). RQ did not significantly differ between the baseline and follow‐up, but there was a significant increase between the baseline and the beginning of the 3‐h measurement (0.76 ± 0.01 vs. 0.80 ± 0.01, *p* < 0.001) and between the end of the 3‐h measurement and follow‐up (0.74 ± 0.01 vs. 0.75 ± 0.01, *p* = 0.010) for both treatments (Figure 5).

#### 4.3.2 Blood pressure (BP) and heart rate (HR)

Systolic blood pressure (SBP) [beginning (15 min) vs. end (180 min) of 3‐h measurement: 109 ± 1 mmHg (both treatments) vs. 111 ± 1 mmHg (xanthohumol) and 110 ± 1 mmHg (placebo)], diastolic blood pressure (DBP) [67 ± 1 mmHg (both treatments) vs. 69 ± 1 mmHg (xanthohumol) and 67 ± 1 mmHg (placebo)], and HR [54 ± 1 vs. 61 ± 1 bpm (both treatments)] all increased during the 3‐h measurement (time effect: all *p* < 0.001). Baseline and follow‐up measurements of SBP, DBP, and HR did not significantly differ. SBP (107 ± 1 vs. 109 ± 1 mmHg (both treatments), *p* = 0.007) and DBP (64 ± 1 vs. 67 ± 1 mmHg (both treatments), *p* < 0.001) significantly increased from the baseline to the beginning of the 3‐h measurement and significantly decreased from the end of the 3‐h measurement to follow‐up (SBP: 111 ± 1 mmHg (xanthohumol); 110 ± 1 mmHg (placebo) vs. 108 ± 1 mmHg (both treatments), *p* < 0.001; DBP: xanthohumol: 68 ± 1 vs. 65 ± 1 mmHg; placebo: 67 ± 1 vs. 64 ± 1 mmHg, *p* < 0.001). HR did not significantly differ between baseline and follow‐up but significantly differed between baseline and the beginning of the 3‐h measurement [56 ± 1 (baseline) vs. 54 ± 1 (15 min) bpm (both treatments)] and between the end of the 3‐h measurement and follow‐up [xanthohumol: 61 ± 1 (180 min) vs. 57 ± 1 (1440 min) bpm; placebo: 61 ± 1 (180 min) vs. 58 ± 1 (1440 min) bpm, *p* < 0.001].

**FIGURE 6:**
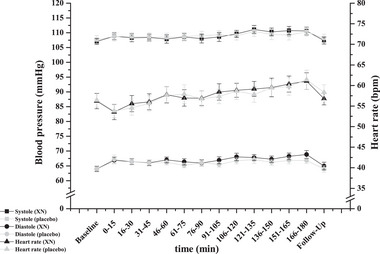
BP and HR time curves in metabolically healthy women after a single oral dose of 172 mg micellar xanthohumol or placebo (*n* = 16). Values represent mean ± SEM.

There was no treatment or treatment × time effect for SBP (treatment: *p* = 0.285, treatment × time: *p* = 0.890), DBP (treatment: *p* = 0.147, treatment × time: *p* = 0.974), or HR (treatment: *p* = 0.712, treatment × time: *p* = 0.939) (Figure 6).

## 5 Discussion

The aim of the present project was to systematically investigate the bioavailability of native xanthohumol compared to micellar xanthohumol at two doses (86 mg vs. 172 mg) in a randomized crossover trial. We furthermore examined the short‐term effects of xanthohumol on REE, BP, and HR in a randomized placebo‐controlled crossover study.

Micellar solubilization significantly increased AUC, *C*_max_, *t*_max_, and apparent bioavailability compared to native xanthohumol. The dose also significantly influenced plasma kinetics, but apparent bioavailability and *t*_max_ were dose‐independent in contrast to AUC and *C*_max_. In our subsequent study, xanthohumol did not affect REE, substrate oxidation, BP, or HR.

To the best of our knowledge, this is the first xanthohumol plasma kinetics study to test the effects of different doses and formulations of xanthohumol in 12 healthy volunteers using a crossover design. Our data indicate that plasma concentrations can be significantly increased by increasing the dose of xanthohumol and by administering micellar xanthohumol. In rats, plasma concentrations of xanthohumol were saturable after administration of higher doses of 40, 100, and 200 mg/kg body weight and could not be further increased [8]. In other human studies, only the influence of the dose of native xanthohumol was tested. However, there is currently no reference method to measure xanthohumol in plasma, which is why the results of different studies are only comparable to a limited extent [9, 11, 32].

After administration of 180 mg native xanthohumol, Legette et al. were able to achieve a mean plasma concentration of 133 ± 23 µg/L (∼375 ± 65 nmol/L), which is comparable to the present study [9]. Van Breemen et al. achieved a lower *C*_max_ of 27.6 ± 8.9 ng/mL (∼0.0779 ± 0.0251 nmol/L) after administration of 85.2 mg xanthohumol [32]. Administration of micellar xanthohumol increased *C*_max_ in Buckett et al. after an administration of 43 mg xanthohumol by approx. 10‐fold to ∼10.6 nmol/L compared to the administration of native xanthohumol [11]. The administration in micellar form at a high dose of 172 mg increased the mean *C*_max_ to 2379 ± 288 nmol/L in the present study.

The elimination half‐life was ∼9–10 h for micellar xanthohumol and 17–25 h for native xanthohumol in the present study. This result is in agreement with other human studies reporting mean elimination half‐lives of total xanthohumol of 16–19 h after an oral dose of 180 mg and 19–20 h after an oral dose of 60 mg native xanthohumol in 48 healthy men and women [9]. Another human study reported mean elimination half‐lives of 18.3 h after a dose of 21.3 mg to 20.7 h at a dose of 85.2 mg of native xanthohumol in 5 postmenopausal women [32]. To determine the elimination half‐life more precisely, excreted metabolites in urine should have been determined. The plasma concentration reflects the systemic availability of xanthohumol, whereas urine concentrations represent the cumulative excretion process.

Similar to previous studies [9, 10, 11, 14, 32, 33], glucuronide was the predominant xanthohumol conjugate in our trial. Xanthohumol accounted for only a very small proportion of total xanthohumol in plasma, which indicates efficient phase II metabolism. The second slight increase in total xanthohumol plasma concentrations occurred ∼120–360 min after administration, depending on the treatment, and is indicative of enterohepatic recirculation (Figure 4).

The measured mean values of 8‐prenylnaringenin were below 0.1 µmol/L for all interventions and showed high interindividual variation (Tables S2–S5). However, due to a lack of reference standards, we were not able to quantify 8‐prenylnaringenin glucuronides and sulfates. Plasma concentrations of 8‐prenylnaringenin higher than 0.1 µmol/L were observed at several different time points, regardless of dose, formulation and subject (Tables S2–S6). Interestingly, our data do not reveal a consistent pattern regarding the conditions under which 8‐prenylnaringenin is formed. In 10 of the 12 participants, irrespective of the intervention, there was at least one time point at which small amounts, or none at all, of 8‐prenylnaringenin were detectable (Tables S2–S5). However, insofar as 8‐prenylnaringenin plasma concentrations could be detected, these were above the values previously detected in human trials. In a pharmacokinetic study administering three different doses of hop extract, the highest dose contained 0.36 mg 8‐prenylnaringenin, 1.86 mg 6‐prenylnaringenin, 1.15 mg isoxanthohumol, and 30.48 mg xanthohumol and resulted in maximum 8‐prenylnaringenin plasma concentrations of 6.7 ± 3.8 nmol/L. In contrast to our study, there was a dose‐dependent increase in the plasma concentration of 8‐prenylnaringenin (Table S6) [32]. High interindividual differences in the transformation of xanthohumol to 8‐prenylnaringenin depending on the composition of the gut microbiome have been reported and might, at least in part, explain the high variability in 8‐prenylnaringenin concentrations observed in our subjects and across human trials [34, 35].

Estrogen receptor alpha transactivation was observed in vitro at a concentration of 8‐prenylnaringenin of 0.029 µmol/L [36]. However, in vitro assays represent simplified systems that do not account for human exposure levels, metabolism, or endocrine feedback mechanisms; consequently, their relevance for direct in vivo health risk assessment is limited. A randomized crossover study with eight healthy postmenopausal women found a linear dose–response relationship in endocrine effects following the administration of 50, 250, or 750 mg of 8‐prenylnaringenin and maximum plasma concentrations at the highest dose of 35 ng/ml (∼0.103 µmol/L). At this dose, a 16.7% reduction in luteinizing hormone was observed with large interindividual differences (95% CI: 0.5–30.2%) [37].

The plasma concentrations of native 8‐prenylnaringenin measured in this study indicate that regular consumption of xanthohumol may lead to steady‐state plasma concentration of 8‐prenylnaringenin. Whether or not these low plasma concentrations may elicit endocrine effects in humans warrants further investigation in randomized, placebo‐controlled trials.

Two properties of xanthohumol impair its bioavailability. First, xanthohumol is relatively unstable in an acidic environment, and second, xanthohumol is highly lipophilic and hydrophobic and thus insoluble in the aqueous environment of the intestinal lumen and poorly absorbed into enterocytes [4]. Strategies to protect against the acidic gastric environment are to administer xanthohumol in the form of a food matrix [33], to use a tertiary fiber membrane as a carrier layer for xanthohumol [38], or to use nanoemulsions, which additionally improve the water solubility of xanthohumol [39, 40]. The cyclization of xanthohumol to isoxanthohumol under the acidic conditions of the stomach [41] could perhaps be reduced using a micellar delivery system, as in the present study. We quantified concentrations of isoxanthohumol and its metabolites in the plasma and found higher *C*_max_ for isoxanthohumol and higher *C*_max_ and AUC for its glucuronide and sulfate metabolites (Table 4), suggesting that more isoxanthohumol was absorbed in the intestine from the micellar formulation. Because isoxanthohumol was quantified in plasma and not in intestinal fluid, it is unknown if the cyclization of xanthohumol to isoxanthohumol in the stomach differed between native and micellar xanthohumol, if the formed isoxanthohumol was more stable in the micelles, and/or if the conversion occurred (partly) post‐absorption, for example, within the lysosomal compartment. This phenomenon warrants further investigation in future experiments.

In the present study, an emulsion process was used with NovaSOL technology to enhance the solubility of xanthohumol using polysorbate 80 (E433) as a component of the micelle. Polysorbate 80 (E433) also improves permeability through the lipophilic intestinal membrane [42]. In a previous study, *C*_max_ increased 20‐fold to ∼200 nmol/L after administration of 43 mg micellar xanthohumol in five healthy adults [11]. In the present study, a higher dose of xanthohumol increased the plasma concentration even further.

In addition to determining typical plasma kinetic parameters (e.g., *C*_max_, *t*_max_, and AUC), we also calculated the amount of xanthohumol absorbed using the maximum plasma xanthohumol concentration (Table 3). These estimated amounts are the minimum quantities of xanthohumol that had to be absorbed to achieve the observed plasma xanthohumol concentrations. The results of these calculations emphasized the relatively poor bioavailability of xanthohumol in humans. Only ∼0.1% and ∼1.2% of native and micellar xanthohumol were absorbed, respectively (Table 3). However, a limitation of this plasma estimation is that fractional absorption of xanthohumol was underestimated because the distribution of xanthohumol in cells and tissues was not taken into account. Thus, it is likely that more xanthohumol was actually absorbed. Whether the bioavailability of xanthohumol is sufficient for physiological efficacy must be investigated further in humans.

In the pharmacokinetic trial, the timepoint at which *C*_max_ was reached appeared to be 1 h, but it is possible that 3 h was too short to detect metabolic effects in the bioactivity study. Furthermore, it seems likely that *C*_max_ was still too low to exert an acute effect on energy metabolism in humans. It is also possible that the effect of xanthohumol on REE is not immediate but delayed, because xanthohumol must first accumulate in the target tissue. This would require xanthohumol to be taken over a longer period of time. In various mouse and rat models, administration of xanthohumol reduces weight [43, 44], weight gain [45], or gain of white adipose tissue [45, 46, 47]. The results obtained from cell and animal model studies suggest that xanthohumol affects body weight by increasing energy metabolism. Although there are indications of an increase in energy metabolism at the cellular level, the exact function of xanthohumol in energy metabolism has not been sufficiently researched. The extent to which xanthohumol directly or indirectly affects BP or HR is also unclear because this is the first study to investigate this issue.

### 5.1 Strengths and Limitations

A strength of the present human trials lies in their designs, namely a randomized, blinded crossover design for the bioavailability study and a placebo‐controlled, double‐blinded crossover design for the bioactivity study. The first was conducted with both sexes, and the menstrual cycle of participants was considered in the latter. Diet and activity were controlled on respective days before measurements in both studies.

Few studies have recorded the plasma kinetics of xanthohumol in humans. Here, we are the first to investigate the influence of formulation and dose in combination. A further strength is the recording of plasma kinetics over 9 h, with a 24‐h follow‐up. In addition, the participants were very homogeneous, comprising young, metabolically healthy adults of both sexes. To minimize further confounding factors, activity was monitored prior to each study day and diet was standardized. A limitation of the bioavailability trial is that double blinding was not possible for study personnel because the appearance of the capsules for two formulations differed and the higher dose was achieved by administering more capsules. In addition, the elimination half‐life could only be averaged over all subjects because the time of the final onset of elimination was very individual and phase II metabolism differed between participants. After 24 h, xanthohumol was still detectable and important timepoints for elimination could no longer be recorded.

## 6 Conclusion

In conclusion, the oral bioavailability of micellar xanthohumol was higher than that of native xanthohumol. The systemic availability of xanthohumol did not differ between men and women. Our study provides no evidence that xanthohumol acutely affects REE, BP, and HR.

## Conflicts of Interest

J.F. is a scientific consultant to AQUANOVA AG and has received honoraria from the company.

## Supporting information




**Supporting File 1**: mnfr70413‐sup‐0001‐FigureS1.pdf.

**Supporting File 2**: mnfr70413‐sup‐0002‐FigureS2.pdf.

**Supporting File 3**: mnfr70413‐sup‐0003‐FigureS3.pdf.

**Supporting File 4**: mnfr70413‐sup‐0004‐TableS1.docx.

**Supporting File 5**: mnfr70413‐sup‐0005‐TableS2.docx.

**Supporting File 6**: mnfr70413‐sup‐0006‐TableS3.docx.

**Supporting File 7**: mnfr70413‐sup‐0007‐TableS4.docx.

**Supporting File 8**: mnfr70413‐sup‐0008‐TableS5.docx.

**Supporting File 9**: mnfr70413‐sup‐0009‐TableS6.docx.

## Data Availability

The data that support the findings of this study are available from the corresponding author upon reasonable request.

## References

[1] P. Dostalek , M. Karabin , and L. Jelinek , “Hop Phytochemicals and Their Potential Role in Metabolic Syndrome Prevention and Therapy,” Molecules 22 (2017): 1761. 29048380 10.3390/molecules22101761 PMC6151408

[2] M. Karabin , T. Hudcova , L. Jelinek , and P. Dostalek , “Biologically Active Compounds From Hops and Prospects for Their Use,” Comprehensive Reviews in Food Science And Food Safety 15 (2016): 542–567. 33401815 10.1111/1541-4337.12201

[3] B. Vanhoecke , L. Derycke , V. Van Marck , H. Depypere , D. De Keukeleire , and M. Bracke , “Antiinvasive Effect of Xanthohumol, a Prenylated Chalcone Present in Hops (*Humulus lupulus* L.) and Beer,” International Journal of Cancer 117 (2005): 889–895. 15986430 10.1002/ijc.21249

[4] E. Oledzka , “Xanthohumol—A Miracle Molecule With Biological Activities: A Review of Biodegradable Polymeric Carriers and Naturally Derived Compounds for Its Delivery,” International Journal of Molecular Sciences 25 (2024): 3398. 38542371 10.3390/ijms25063398 PMC10970401

[5] L. Zhang , J. Lv , W. Zhang , et al., “Functionalized Xanthohumol Nanoemulsion: Fabrication, Characterization and Bioavailability Enhancement of Bioactive Compounds,” Journal of the Science of Food and Agriculture 104 (2024): 9442–9450. 39082082 10.1002/jsfa.13767

[6] M. T. Khayyal , R. M. El‐Hazek , W. A. El‐Sabbagh , J. Frank , D. Behnam , and M. Abdel‐Tawab , “Micellar Solubilization Enhances the Anti‐Inflammatory Effect of Xanthohumol,” Phytomedicine 71 (2020): 153233. 32454348 10.1016/j.phymed.2020.153233

[7] L. Legette , L. Ma , R. L. Reed , et al., “Pharmacokinetics of Xanthohumol and Metabolites in Rats After Oral and Intravenous Administration,” Molecular Nutrition & Food Research 56 (2012): 466–474. 22147307 10.1002/mnfr.201100554 PMC3401605

[8] B. Nowak , B. Pozniak , J. Poplonski , et al., “Pharmacokinetics of Xanthohumol in Rats of Both Sexes After Oral and Intravenous Administration of Pure Xanthohumol and Prenylflavonoid Extract,” Advances in Clinical and Experimental Medicine: Official Organ Wroclaw Medical University 29 (2020): 1101–1109. 32996724 10.17219/acem/126293

[9] L. Legette , C. Karnpracha , R. L. Reed , et al., “Human Pharmacokinetics of Xanthohumol, an Antihyperglycemic Flavonoid From Hops,” Molecular Nutrition & Food Research 58 (2014): 248–255. 24038952 10.1002/mnfr.201300333 PMC4371792

[10] S. Bolca , J. Li , D. Nikolic , et al., “Disposition of Hop Prenylflavonoids in Human Breast Tissue,” Molecular Nutrition & Food Research 54 Suppl 2 (2010): S284–S294. 20486208 10.1002/mnfr.200900519 PMC3856213

[11] L. Buckett , N. Sus , V. Spindler , M. Rychlik , C. Schoergenhofer , and J. Frank , “The Pharmacokinetics of Individual Conjugated Xanthohumol Metabolites Show Efficient Glucuronidation and Higher Bioavailability of Micellar Than Native Xanthohumol in a Randomized, Double‐Blind, Crossover Trial in Healthy Humans,” Molecular Nutrition & Food Research 67 (2023): 220068. 10.1002/mnfr.202200684 37721120

[12] F. Ferk , M. Misik , A. Nersesyan , et al., “Impact of Xanthohumol (a Prenylated Flavonoid From Hops) on DNA Stability and Other Health‐Related Biochemical Parameters: Results of Human Intervention Trials,” Molecular Nutrition & Food Research 60 (2016): 773–786. 26840505 10.1002/mnfr.201500355

[13] R. Bradley , B. O. Langley , J. J. Ryan , et al., “Xanthohumol Microbiome and Signature in Healthy Adults (the XMaS Trial): A Phase I Triple‐Masked, Placebo‐Controlled Clinical Trial,” Trials 21 (2020): 835. 33028396 10.1186/s13063-020-04769-2 PMC7542976

[14] B. O. Langley , J. J. Ryan , D. Hanes , et al., “Xanthohumol Microbiome and Signature in Healthy Adults (the XMaS Trial): Safety and Tolerability Results of a Phase I Triple‐Masked, Placebo‐Controlled Clinical Trial,” Molecular Nutrition & Food Research 65 (2021): 2001170. 10.1002/mnfr.202001170 PMC8221389 33629812

[15] H. F. Neumann , J. Frank , S. Venturelli , and S. Egert , “Bioavailability and Cardiometabolic Effects of Xanthohumol: Evidence From Animal and Human Studies,” Molecular Nutrition & Food Research 66 (2022): 2100831. 10.1002/mnfr.202100831 34967501

[16] S. P. Gómez‐Zorita , A. Fernández‐Quintela , I. Moreno‐Indias , and M. P. Portillo , “Beneficial Effects of Xanthohumol on Metabolic Syndrome: Evidence From In Vitro and Animal Model Studies,” International Journal of Molecular Sciences 25 (2024): 124–134. 10.3390/ijms252212434 PMC11594861 39596505

[17] M. S. Merkel and K. A. Iwen , “Physiologie und Klinische Bedeutung von Weißem, Beigem und Braunem Fettgewebe,” Internist 60 (2019): 115–121. 30617700 10.1007/s00108-018-0540-0

[18] P. Bostrom , J. Wu , M. P. Jedrychowski , et al., “A PGC1‐α‐Dependent Myokine That Drives Brown‐Fat‐Like Development of White Fat and Thermogenesis,” Nature 481 (2012): 463–468. 22237023 10.1038/nature10777 PMC3522098

[19] A. B. Iniguez and M. J. Zhu , “Hop Bioactive Compounds in Prevention of Nutrition‐Related Noncommunicable Diseases,” Critical Reviews in Food Science and Nutrition 61 (2021): 1900–1913. 32462886 10.1080/10408398.2020.1767537

[20] N. Sus , J. Schlienz , A. Laura , et al., “Validation of a Rapid and Sensitive Reversed‐Phase Liquid Chromatographic Method for the Quantification of Prenylated Chalcones and Flavanones in Plasma and Urine,” NFS Journal 10 (2018): 1–9.

[21] L. Buckett , S. Schonberger , V. Spindler , et al., “Synthesis of Human Phase I and Phase II Metabolites of Hop (*Humulus lupulus*) Prenylated Flavonoids,” Metabolites 12 (2022): 345. 35448532 10.3390/metabo12040345 PMC9030851

[22] B. E. Ainsworth , W. L. Haskell , M. C. Whitt , et al., “Compendium of Physical Activities: An Update of Activity Codes and MET Intensities,” Medicine and Science in Sports and Exercise 32 (2000): S498–S504. 10993420 10.1097/00005768-200009001-00009

[23] S. S. Sun , W. C. Chumlea , S. B. Heymsfield , et al., “Development of Bioelectrical Impedance Analysis Prediction Equations for Body Composition With the Use of a Multicomponent Model for Use in Epidemiologic Surveys,” American Journal of Clinical Nutrition 77 (2003): 331–340. 12540391 10.1093/ajcn/77.2.331

[24] S. B. Nadler , J. H. Hidalgo , and T. Bloch , “Prediction of Blood Volume in Normal Human Adults,” Surgery 51 (1962): 224–232. 21936146

[25] Guidance for Industry: Statistical Approaches to Establishing Bioequivalence (Food and Drug Administration, 2001).

[26] Guideline on the Investigation of Bioequivalence (European Medicines Agency, 2010). 10.1111/j.1742-7843.2009.00518.x 20070293

[27] C. Burak , V. Brull , P. Langguth , et al., “Higher Plasma Quercetin Levels Following Oral Administration of an Onion Skin Extract Compared With Pure Quercetin Dihydrate in Humans,” European Journal of Nutrition 56 (2017): 343–353. 26482244 10.1007/s00394-015-1084-x

[28] J. B. Weir , “New Methods for Calculating Metabolic Rate With Special Reference to Protein Metabolism,” Journal of Physiology 109 (1949): 1–9. 15394301 10.1113/jphysiol.1949.sp004363 PMC1392602

[29] P. K. Whelton , R. M. Carey , W. S. Aronow , et al., “ACC/AHA/AAPA/ABC/ACPM/AGS/APhA/ASH/ASPC/NMA/PCNA Guideline for the Prevention, Detection, Evaluation, and Management of High Blood Pressure in Adults: Executive Summary: A Report of the American College of Cardiology/American Heart Association Task Force on Clinical Practice Guidelines,” Hypertension 71 (2018): 1269–1324. 29133354 10.1161/HYP.0000000000000066

[30] B. Williams , G. Mancia , W. Spiering , et al., “2018 ESC/ESH Guidelines for the Management of Arterial Hypertension,” European Heart Journal 39 (2018): 3021–3104. 30165516 10.1093/eurheartj/ehy339

[31] S. Riva‐Rocci , “Un Nuovo Sfigmomanometro,” Gazz Med Torino 47 (1896): 981–1001.

[32] R. B. van Breemen , Y. Yuan , S. Banuvar , et al., “Pharmacokinetics of Prenylated Hop Phenols in Women Following Oral Administration of a Standardized Extract of Hops,” Molecular Nutrition & Food Research 58 (2014): 1962–1969. 25045111 10.1002/mnfr.201400245 PMC4265473

[33] A. O'Connor , V. Konda , R. L. Reed , J. M. Christensen , J. F. Stevens , and N. Contractor , “Rice Protein Matrix Enhances Circulating Levels of Xanthohumol Following Acute Oral Intake of Spent Hops in Humans,” Molecular Nutrition & Food Research 62 (2018): 1700692. 10.1002/mnfr.201700692 29322620

[34] M. Stevanoska , M. Cremona , K. Beekmann , S. J. Sturla , and G. Aichinger , “Interindividual Variation in Gut Microbial Formation of 8‐Prenylnaringenin Results in Increased, but Sub‐Estrogenic, Internal Exposure,” Research Square (2025), 10.21203/rs.3.rs-6511068/v1.

[35] M. Stevanoska , K. Beekmann , A. Punt , S. J. Sturla , and G. Aichinger , “Predicting in Vivo Concentrations of Dietary Hop Phytoestrogens by Physiologically Based Kinetic Modeling,” Food and Chemical Toxicology 196 (2025): 115247. 39793946 10.1016/j.fct.2025.115247

[36] K. S. Sim , S. Park , H. Seo , et al., “Comparative Study of Estrogenic Activities of Phytoestrogens Using OECD In Vitro and In Vivo Testing Methods,” Toxicology and Applied Pharmacology 434 (2022): 115815. 34848279 10.1016/j.taap.2021.115815

[37] M. Rad , M. Humpel , O. Schaefer , et al., “Pharmacokinetics and Systemic Endocrine Effects of the Phyto‐Oestrogen 8‐Prenylnaringenin After Single Oral Doses to Postmenopausal Women,” British Journal of Clinical Pharmacology 62 (2006): 288–296. 16934044 10.1111/j.1365-2125.2006.02656.x PMC1885137

[38] T. Qiao , S. Jiang , P. Song , et al., “Effect of Blending HA‐g‐PLLA on Xanthohumol‐Loaded PLGA Fiber Membrane,” Colloids and Surfaces B: Biointerfaces 146 (2016): 221–227. 27343844 10.1016/j.colsurfb.2016.06.011

[39] V. Harish , D. Tewari , S. Mohd , et al., “Quality by Design Based Formulation of Xanthohumol Loaded Solid Lipid Nanoparticles With Improved Bioavailability and Anticancer Effect Against PC‐3 Cells,” Pharmaceutics 14 (2022): 2403. 36365221 10.3390/pharmaceutics14112403 PMC9699314

[40] M. Hanmantrao , S. Chaterjee , R. Kumar , et al., “Development of Guar Gum‐Pectin‐Based Colon Targeted Solid Self‐Nanoemulsifying Drug Delivery System of Xanthohumol,” Pharmaceutics 14 (2022): 2384. 36365203 10.3390/pharmaceutics14112384 PMC9693267

[41] D. Nikolic , Y. Li , L. R. Chadwick , G. F. Pauli , and R. B. van Breemen , “Metabolism of Xanthohumol and isoxanthohumol, Prenylated Flavonoids From Hops (*Humulus lupulus* L.), by Human Liver Microsomes,” Journal of Mass Spectrometry 40 (2005): 289–299. 15712367 10.1002/jms.753

[42] J. B. Cannon , “Strategies to Formulate Lipid‐Based Drug Delivery Systems,” American Pharmaceutical Review 14 (2011): 84.

[43] L. L. Legette , A. Y. Luna , R. L. Reed , et al., “Xanthohumol Lowers Body Weight and Fasting Plasma Glucose in Obese Male Zucker Fa/Fa Rats,” Phytochemistry 91 (2013): 236–241. 22640929 10.1016/j.phytochem.2012.04.018

[44] C. L. Miranda , V. D. Elias , J. J. Hay , J. Choi , R. L. Reed , and J. F. Stevens , “Xanthohumol Improves Dysfunctional Glucose and Lipid Metabolism in Diet‐Induced Obese C57BL/6J Mice,” Archives of Biochemistry and Biophysics 599 (2016): 22–30. 26976708 10.1016/j.abb.2016.03.008 PMC4875845

[45] A. Mahli , T. Seitz , K. Freese , et al., “Therapeutic Application of Micellar Solubilized Xanthohumol in a Western‐Type Diet‐Induced Mouse Model of Obesity, Diabetes and Non‐Alcoholic Fatty Liver Disease,” Cells 8 (2019): 359. 30999670 10.3390/cells8040359 PMC6523748

[46] H. Nozawa , “Xanthohumol, the Chalcone From Beer Hops (*Humulus lupulus* L.), is the Ligand for Farnesoid X Receptor and Ameliorates Lipid and Glucose Metabolism in KK‐A Mice,” Biochemical and Biophysical Research Communications 336 (2005): 754–761. 16140264 10.1016/j.bbrc.2005.08.159

[47] A. K. Hamm , D. K. Manter , J. S. Kirkwood , L. M. Wolfe , K. Cox‐York , and T. L. Weir , “The Effect of Hops (*Humulus lupulus* L.) Extract Supplementation on Weight Gain, Adiposity and Intestinal Function in Ovariectomized Mice,” Nutrients 11 (2019): 3004. 31817899 10.3390/nu11123004 PMC6950254

