# Supplementary figures and images for: The Bioavailability of Xanthohumol in Humans and the Influence of Formulation and Dose: Randomized Controlled Trial Data
Source: Mol Nutr Food Res. 2026 Feb 22;70(4):e70413. doi: 10.1002/mnfr.70413 (PMC12925386; doi:10.1002/mnfr.70413)

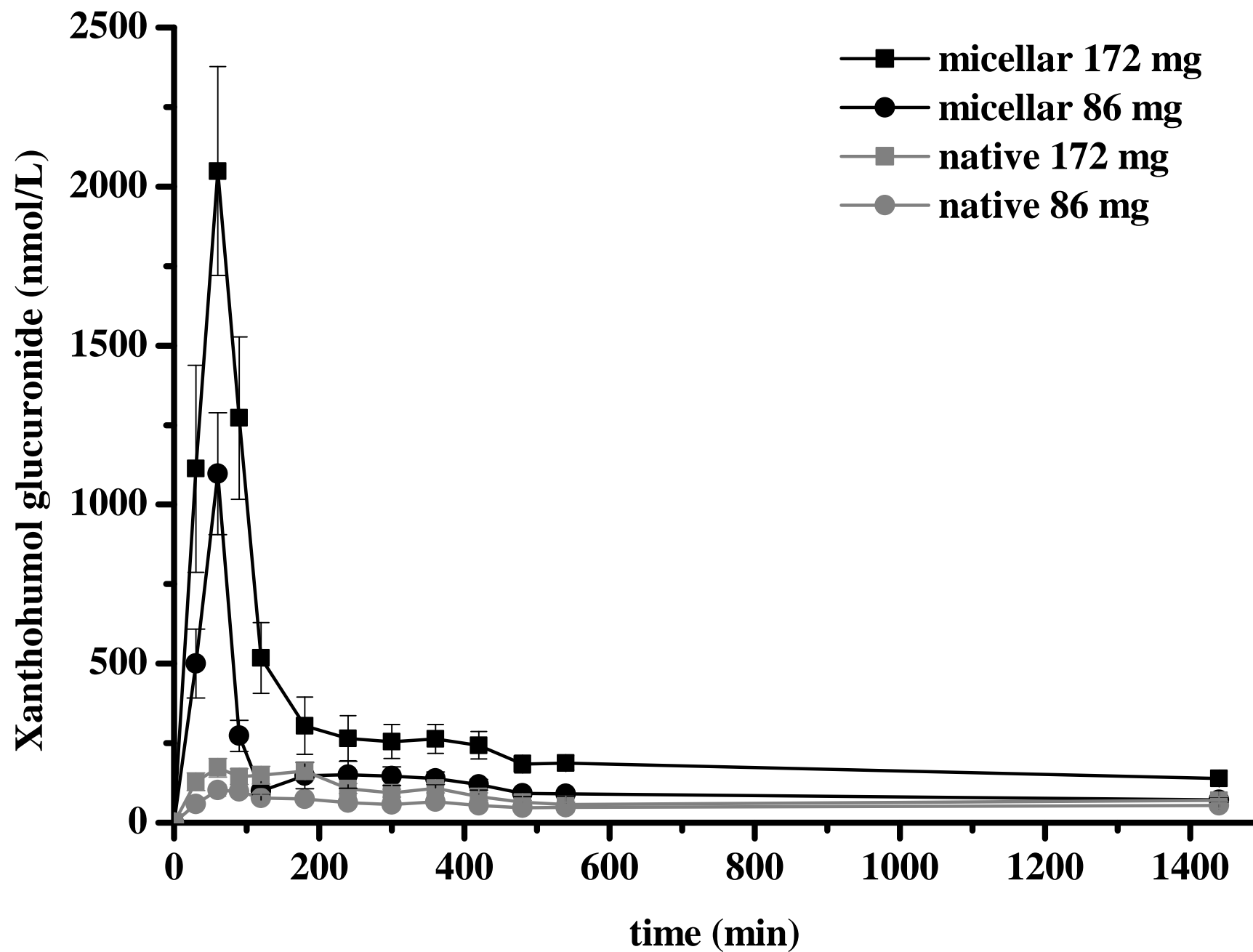

Supplement: Supplementary file 1 — Supporting File 1: mnfr70413‐sup‐0001‐FigureS1.pdf. [file MNFR-70-e70413-s001.pdf]

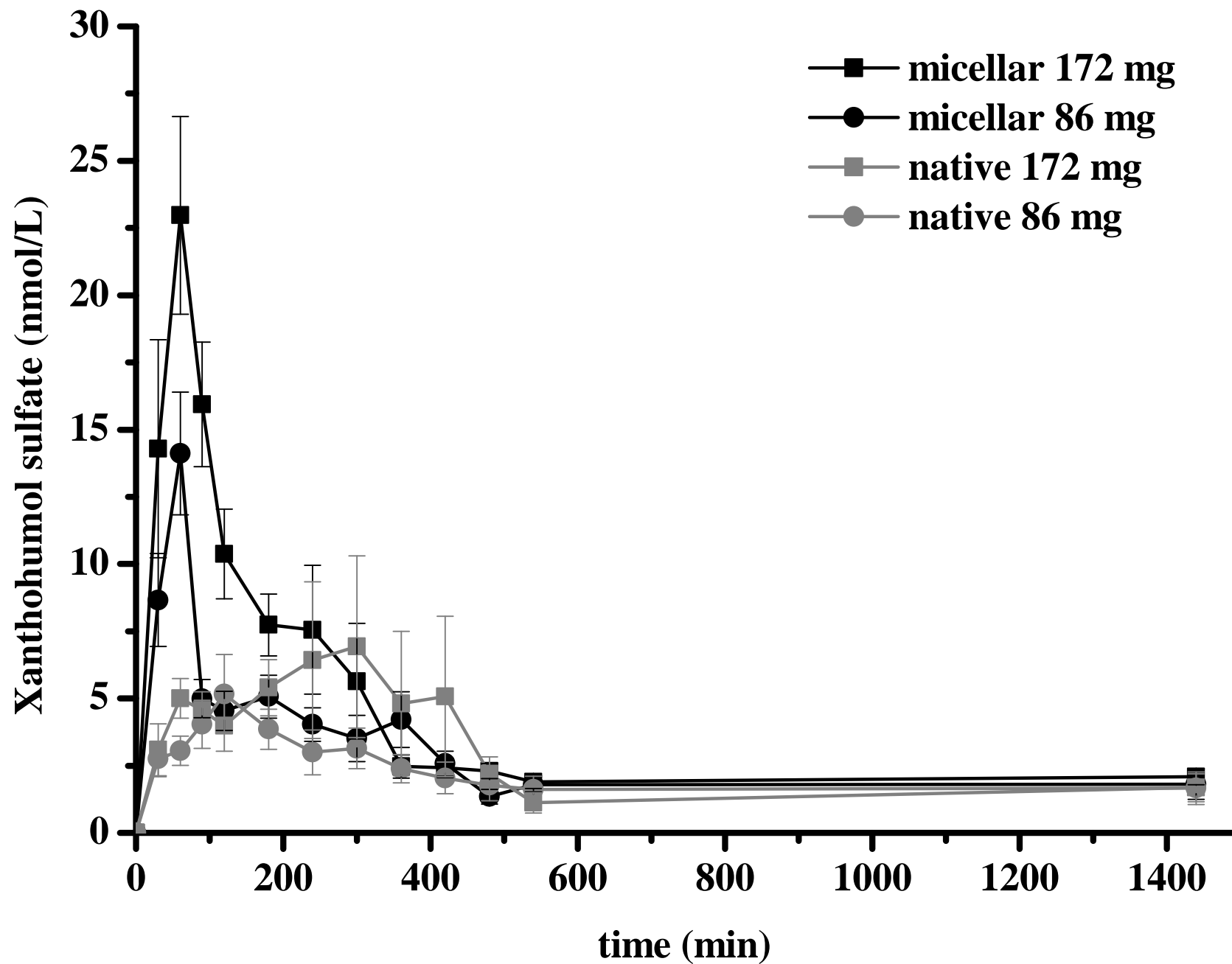

Supplement: Supplementary file 2 — Supporting File 2: mnfr70413‐sup‐0002‐FigureS2.pdf. [file MNFR-70-e70413-s002.pdf]

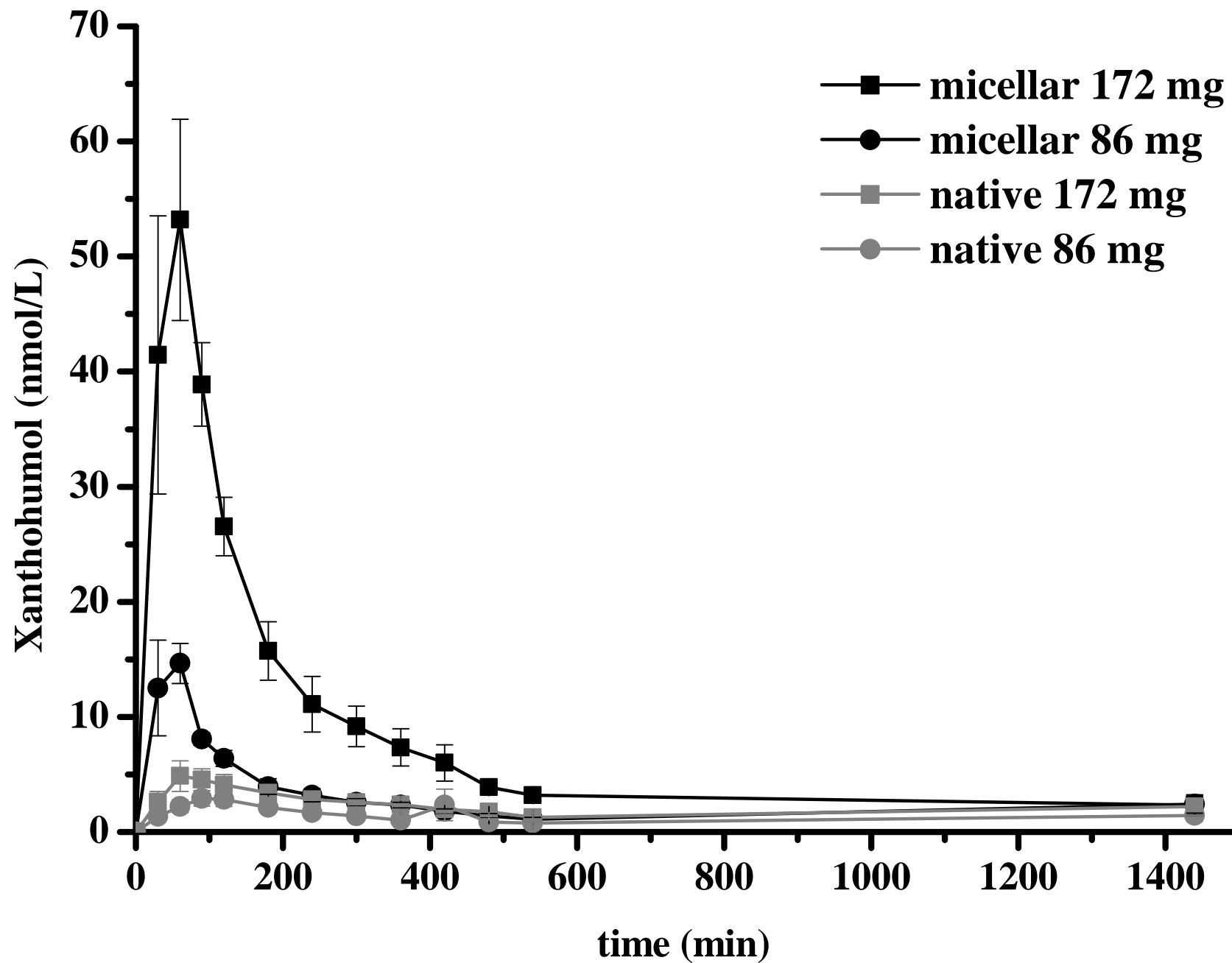

Supplement: Supplementary file 3 — Supporting File 3: mnfr70413‐sup‐0003‐FigureS3.pdf. [file MNFR-70-e70413-s006.pdf]
