# Supplementary material for: The Bioavailability of Xanthohumol in Humans and the Influence of Formulation and Dose: Randomized Controlled Trial Data
Source: Mol Nutr Food Res. 2026 Feb 22;70(4):e70413. doi: 10.1002/mnfr.70413 (PMC12925386; doi:10.1002/mnfr.70413)
Supplement: Supplementary file 4 — Supporting File 4: mnfr70413‐sup‐0004‐TableS1.docx. [file MNFR-70-e70413-s008.docx]

**Supplemental Table 1:** Control parameters on different study visits (bioavailability trial)

|  | Visit 1 (n=12) | Visit 2 (n=12) | Visit 3 (n=12) | Visit 4 (n=12) |
| --- | --- | --- | --- | --- |
| Body weight (kg) | 64 ± 6 | 64 ± 6 | 64 ± 6 | 64 ± 6 |
| FFM (kg) | 48 ± 6 | 49 ± 7 | 49 ± 6 | 49 ± 6 |
| FM (kg) | 15 ± 4 | 15 ± 4 | 15 ± 4 | 15 ± 3 |
| FM (%) | 24 ± 6 | 23 ± 7 | 23 ± 6 | 23 ± 5 |
| Resting SBP (mmHg) | 128 ± 16 | 129 ± 11 | 125 ± 11 | 123 ± 11 |
| Resting DBP (mmHg) | 80 ± 7 | 75 ± 9 | 78 ± 8 | 75 ± 9 |
| Resting pulse rate (bpm) | 67 ± 15 | 75 ± 12 | 68 ± 10 | 67 ± 12 |
| Activity (METs) | 1.5 ± 0.3 | 1.5 ± 0.4 | 1.6 ± 0.3 | 1.6 ± 0.3 |
| Energy intake (kcal/d) | 1943 ± 620 | 2098 ± 575 | 2207 ± 840 | 2184 ± 694 |
| Fat intake (E%) | 36 ± 9 | 37 ± 10 | 39 ± 6 | 39 ± 7 |
| Carbohydrate intake (E%) | 47 ± 10 | 47 ± 10 | 43 ± 7 | 45 ± 10 |
| Protein intake (E%) | 17 ± 3 | 16 ± 3 | 18 ± 5 | 16 ± 4 |
| SAFA (E%) | 13 ± 4 | 14 ± 6 | 17 ± 4 | 15 ± 4 |
| MUFA (E%) | 16 ± 5 | 16 ± 5 | 16 ± 5 | 17 ± 6 |
| PUFA (E%) | 6 ± 3 | 7 ± 3 | 7 ± 2 | 6 ± 3 |
| Dietary fiber (g/1000 kcal) | 12 ± 5 | 14 ± 4 | 12 ± 6 | 12 ± 5 |

Data represent mean ± SD. FFM, fat-free mass; FM, fat mass; SBP, systolic blood pressure; DBP, diastolic blood pressure; METS, metabolic equivalent of tasks; SAFA, saturated fatty acid
