# Supplementary material for: The Bioavailability of Xanthohumol in Humans and the Influence of Formulation and Dose: Randomized Controlled Trial Data
Source: Mol Nutr Food Res. 2026 Feb 22;70(4):e70413. doi: 10.1002/mnfr.70413 (PMC12925386; doi:10.1002/mnfr.70413)
Supplement: Supplementary file 5 — Supporting File 5: mnfr70413‐sup‐0005‐TableS2.docx. [file MNFR-70-e70413-s009.docx]

**Supplemental Table 2:** Plasma concentration of 8-prenylnaringenin of n = 12 participants after oral ingestion of 86 mg native xanthohumol

| **Subject pseudonym/**  **min** | **0** | **30** | **60** | **90** | **120** | **180** | **240** | **300** | **360** | **420** | **480** | **540** | **1440** |
| --- | --- | --- | --- | --- | --- | --- | --- | --- | --- | --- | --- | --- | --- |
| **Tf** | n.d. | n.d. | n.d. | n.d. | n.d. | n.d. | n.d. | n.d. | n.d. | n.d. | n.d. | n.d. | n.d. |
| **Lb** | n.d. | n.d. | n.d. | n.d. | n.d. | n.d. | n.d. | n.d. | n.d. | n.d. | n.d. | n.d. | n.d. |
| **Qp** | n.d. | n.d. | n.d. | n.d. | n.d. | n.d. | n.d. | n.d. | n.d. | n.d. | n.d. | n.d. | n.d. |
| **Nd** | n.d. | n.d. | n.d. | n.d. | n.d. | n.d. | n.d. | n.d. | n.d. | n.d. | n.d. | n.d. | n.d. |
| **Sy** | n.d. | n.d. | n.d. | n.d. | n.d. | n.d. | n.d. | n.d. | n.d. | n.d. | n.d. | n.d. | n.d. |
| **Jm** | n.d. | n.d. | n.d. | n.d. | 24 | 21 | n.d. | n.d. | n.d. | n.d. | n.d. | n.d. | n.d. |
| **Ap** | n.d. | n.d. | n.d. | n.d. | 69 | 392 | 51 | 236 | 161 | 119 | 382 | 49 | 66 |
| **Rk** | n.d. | 71 | 43 | 104 | 107 | 139 | 124 | 134 | 120 | 103 | n.d. | n.d. | n.d. |
| **Cw** | n.d. | 33 | 26 | 8 | n.d. | n.d. | n.d. | n.d. | n.d. | n.d. | n.d. | n.d. | n.d. |
| **Ge** | n.d. | n.d. | 16 | 17 | 9 | n.d. | n.d. | n.d. | n.d. | n.d. | n.d. | n.d. | n.d. |
| **Xh** | n.d. | 1 | n.d. | n.d. | n.d. | n.d. | n.d. | n.d. | n.d. | n.d. | n.d. | n.d. | n.d. |
| **Zv** | n.d. | n.d. | n.d. | n.d. | n.d. | n.d. | 8 | 13 | 6 | 4 | 6 | n.d. | n.d. |

Data represent absolute values of native 8-prenylnaringenin plasma concentration in nmol/L. n.d., not detectable.
