# Supplementary material for: The Bioavailability of Xanthohumol in Humans and the Influence of Formulation and Dose: Randomized Controlled Trial Data
Source: Mol Nutr Food Res. 2026 Feb 22;70(4):e70413. doi: 10.1002/mnfr.70413 (PMC12925386; doi:10.1002/mnfr.70413)
Supplement: Supplementary file 6 — Supporting File 6: mnfr70413‐sup‐0006‐TableS3.docx. [file MNFR-70-e70413-s003.docx]

**Supplemental Table 3:** Plasma concentration of 8-prenylnaringenin of n = 12 participants after oral ingestion of 172 mg native xanthohumol

| **Subject pseudonym/**  **min** | **0** | **30** | **60** | **90** | **120** | **180** | **240** | **300** | **360** | **420** | **480** | **540** | **1440** |
| --- | --- | --- | --- | --- | --- | --- | --- | --- | --- | --- | --- | --- | --- |
| **Tf** | n.d. | n.d. | n.d. | n.d. | n.d. | n.d. | n.d. | n.d. | n.d. | n.d. | n.d. | n.d. | n.d. |
| **Lb** | n.d. | 66 | 59 | 2 | 31 | 17 | 2n.d. | 4 | 13 | 15 | 34 | n.d. | n.d. |
| **Qp** | n.d. | n.d. | 8 | 11 | n.d. | n.d. | n.d. | n.d. | n.d. | n.d. | n.d. | n.d. | n.d. |
| **Nd** | n.d. | 14 | 25 | 27 | 25 | 13 | 9 | 6 | n.d. | n.d. | n.d. | n.d. | n.d. |
| **Sy** | n.d. | n.d. | n.d. | n.d. | 3 | 55 | 7 | 19 | 4 | 12 | 13 | 6 | 9 |
| **Jm** | n.d. | n.d. | n.d. | n.d. | n.d. | n.d. | n.d. | n.d. | n.d. | n.d. | n.d. | n.d. | n.d. |
| **Ap** | n.d. | n.d. | n.d. | n.d. | n.d. | n.d. | n.d. | n.d. | n.d. | n.d. | n.d. | n.d. | n.d. |
| **Rk** | n.d. | n.d. | n.d. | n.d. | n.d. | n.d. | 52 | 45 | n.d. | n.d. | n.d. | n.d. | n.d. |
| **Cw** | n.d. | n.d. | n.d. | n.d. | n.d. | n.d. | 4 | n.d. | n.d. | n.d. | n.d. | n.d. | n.d. |
| **Ge** | n.d. | n.d. | n.d. | n.d. | 2 | 47 | 104 | 140 | 90 | 105 | 70 | 48 | 45 |
| **Xh** | n.d. | n.d. | 83 | 71 | n.d. | n.d. | n.d. | n.d. | n.d. | n.d. | n.d. | n.d. | n.d. |
| **Zv** | n.d. | n.d. | n.d. | n.d. | n.d. | n.d. | n.d. | 386 | 117 | 42 | 48 | 19 | n.d. |

Data represent absolute values of native 8-prenylnaringenin plasma concentration in nmol/L. n.d., not detectable.
