# Supplementary material for: The Bioavailability of Xanthohumol in Humans and the Influence of Formulation and Dose: Randomized Controlled Trial Data
Source: Mol Nutr Food Res. 2026 Feb 22;70(4):e70413. doi: 10.1002/mnfr.70413 (PMC12925386; doi:10.1002/mnfr.70413)
Supplement: Supplementary file 7 — Supporting File 7: mnfr70413‐sup‐0007‐TableS4.docx. [file MNFR-70-e70413-s007.docx]

**Supplemental Table 4:** Plasma concentration of 8-Prenylnaringenin of n = 12 participants after an oral ingestion of 86 mg micellar xanthohumol

| **Subject pseudonym/**  **min** | **0** | **30** | **60** | **90** | **120** | **180** | **240** | **300** | **360** | **420** | **480** | **540** | **1440** |
| --- | --- | --- | --- | --- | --- | --- | --- | --- | --- | --- | --- | --- | --- |
| **Tf** | n.d. | n.d. | n.d. | 167 | 165 | 161 | 152 | 138 | 119 | 82 | 76 | 62 | n.d. |
| **Lb** | n.d. | n.d. | n.d. | n.d. | 2 | 29 | 46 | 450 | n.d. | n.d. | n.d. | n.d. | n.d. |
| **Qp** | n.d. | n.d. | n.d. | n.d. | n.d. | n.d. | n.d. | n.d. | n.d. | n.d. | n.d. | n.d. | n.d. |
| **Nd** | n.d. | 10 | 1 | 1 | 35 | 1 | n.d. | n.d. | n.d. | n.d. | n.d. | n.d. | n.d. |
| **Sy** | n.d. | n.d. | n.d. | n.d. | n.d. | n.d. | n.d. | n.d. | n.d. | n.d. | n.d. | n.d. | n.d. |
| **Jm** | n.d. | n.d. | n.d. | n.d. | n.d. | n.d. | 124 | 120 | 71 | 14 | 4 | n.d. | n.d. |
| **Ap** | n.d. | n.d. | n.d. | n.d. | n.d. | n.d. | n.d. | n.d. | n.d. | n.d. | n.d. | n.d. | n.d. |
| **Rk** | n.d. | n.d. | n.d. | 84 | n.d. | n.d. | n.d. | n.d. | n.d. | n.d. | n.d. | n.d. | n.d. |
| **Cw** | n.d. | n.d. | n.d. | n.d. | n.d. | n.d. | n.d. | n.d. | n.d. | n.d. | n.d. | n.d. | n.d. |
| **Ge** | n.d. | n.d. | n.d. | n.d. | n.d. | n.d. | n.d. | n.d. | n.d. | n.d. | n.d. | n.d. | n.d. |
| **Xh** | n.d. | 273 | 257 | n.d. | n.d. | n.d. | n.d. | n.d. | n.d. | n.d. | n.d. | n.d. | n.d. |
| **Zv** | n.d. | n.d. | 24 | 16 | 19 | n.d. | n.d. | n.d. | n.d. | n.d. | n.d. | n.d. | n.d. |

Data represent absolute values of native 8-prenylnaringenin plasma concentration in nmol/L. n.d., not detectable.
