# Supplementary material for: The Bioavailability of Xanthohumol in Humans and the Influence of Formulation and Dose: Randomized Controlled Trial Data
Source: Mol Nutr Food Res. 2026 Feb 22;70(4):e70413. doi: 10.1002/mnfr.70413 (PMC12925386; doi:10.1002/mnfr.70413)
Supplement: Supplementary file 8 — Supporting File 8: mnfr70413‐sup‐0008‐TableS5.docx. [file MNFR-70-e70413-s005.docx]

**Supplemental Table 5:** Plasma concentration of 8-Prenylnaringenin of n = 12 participants after an oral ingestion of 172 mg micellar xanthohumol

| **Subject pseudonym/**  **min** | **0** | **30** | **60** | **90** | **120** | **180** | **240** | **300** | **360** | **420** | **480** | **540** | **1440** |
| --- | --- | --- | --- | --- | --- | --- | --- | --- | --- | --- | --- | --- | --- |
| **Tf** | n.d. | n.d. | 59 | 57 | 49 | 48 | 39 | 34 | 34 | 26 | 25 | 31 | n.d. |
| **Lb** | n.d. | 235 | 190 | 74 | n.d. | n.d. | n.d. | n.d. | n.d. | n.d. | n.d. | n.d. | n.d. |
| **Qp** | n.d. | n.d. | 12 | 13 | n.d. | n.d. | n.d. | n.d. | n.d. | n.d. | n.d. | n.d. | n.d. |
| **Nd** | n.d. | n.d. | n.d. | n.d. | n.d. | n.d. | n.d. | n.d. | n.d. | n.d. | n.d. | n.d. | n.d. |
| **Sy** | n.d. | n.d. | n.d. | n.d. | n.d. | n.d. | n.d. | n.d. | n.d. | n.d. | n.d. | n.d. | n.d. |
| **Jm** | n.d. | n.d. | n.d. | n.d. | n.d. | n.d. | 36 | 58 | n.d. | n.d. | n.d. | n.d. | n.d. |
| **Ap** | n.d. | n.d. | n.d. | n.d. | n.d. | n.d. | n.d. | n.d. | n.d. | n.d. | n.d. | n.d. | n.d. |
| **Rk** | n.d. | n.d. | n.d. | n.d. | 3 | 9 | n.d. | n.d. | n.d. | n.d. | n.d. | n.d. | n.d. |
| **Cw** | n.d. | n.d. | n.d. | n.d. | n.d. | n.d. | n.d. | n.d. | n.d. | n.d. | n.d. | n.d. | n.d. |
| **Ge** | n.d. | n.d. | n.d. | n.d. | n.d. | n.d. | n.d. | n.d. | n.d. | n.d. | n.d. | n.d. | n.d. |
| **Xh** | n.d. | n.d. | n.d. | 223 | 217 | n.d. | n.d. | n.d. | n.d. | n.d. | n.d. | n.d. | n.d. |
| **Zv** | n.d. | n.d. | 125 | 115 | 120 | 121 | 169 | 190 | n.d. | n.d. | n.d. | 182 | n.d. |

Data represent absolute values of native 8-prenylnaringenin plasma concentration in nmol/L. n.d., not detectable.
