# Supplementary material for: The Bioavailability of Xanthohumol in Humans and the Influence of Formulation and Dose: Randomized Controlled Trial Data
Source: Mol Nutr Food Res. 2026 Feb 22;70(4):e70413. doi: 10.1002/mnfr.70413 (PMC12925386; doi:10.1002/mnfr.70413)
Supplement: Supplementary file 9 — Supporting File 9: mnfr70413‐sup‐0009‐TableS6.docx. [file MNFR-70-e70413-s004.docx]

**Supplemental Table 6:** Overview of detected 8-prenylnaringenin plasma concentrations of n = 12 participants after an oral ingestion of 86 or 172 mg native xanthohumol or 86 or 172 mg micellar xanthohumol.

|  | **86 mg native xanthohumol** | **172 mg native xanthohumol** | **86 mg micellar xanthohumol** | **172 mg micellar xanthohumol** |
| --- | --- | --- | --- | --- |
| **Number detectable cases** | 32/156 | 46/156 | 29/156 | 28/156 |
| ***C_max_* (nmol/L)** | 392 | 386 | 450 | 235 |
| ***C_min_* (nmol/L)** | 1 | 2 | 1 | 3 |
| ***C_mean_* (nmol/L)** | 83 | 44 | 93 | 89 |
| **SD (*C_mean_*) (nmol/L)** | 97 | 61 | 100 | 73 |
| **SEM (*C_mean_*) (nmol/L)** | 17 | 9 | 19 | 14 |
| **95% Confidence interval (nmol/L)** | 48; 119 | 26; 62 | 54; 132 | 60; 118 |

Number detectable cases are detectable cases compared to potential maximum number of detectable cases (n=156). *C_max_*, highest measured concentration of 8-prenylnaringenin. *C_min_*, lowest measured concentration of 8-prenylnaringenin. *C_mean_*, mean concentration of 8-prenylnaringenin. Mean concentration is calculated as sum of all detectable concentrations divided per number of detectable cases. SD, standard deviation of mean concentration. SEM, standard error of mean of mean concentration.
